# Oestrogen treatment restores dentate gyrus development in premature newborns by IGF1 regulation

**DOI:** 10.1111/jcmm.17816

**Published:** 2023-08-18

**Authors:** Deep R. Sharma, Bokun Cheng, Rauhin Sahu, Xusheng Zhang, Rana Mehdizadeh, Divya Singh, Dumitru Iacobas, Praveen Ballabh

**Affiliations:** ^1^ Department of Pediatrics Albert Einstein College of Medicine Bronx New York USA; ^2^ Computational Genomics Core Albert Einstein College of Medicine Bronx New York USA; ^3^ Dominick P. Purpura Department of Neuroscience Albert Einstein College of Medicine Bronx New York USA; ^4^ Personalized Genomics Laboratory, Texas Undergraduate Medical Academy Prairie View A&M University Prairie View Texas USA

**Keywords:** dentate gyrus, IGF1, oestrogen and oestrogen receptor α, prematurity

## Abstract

Prematurely‐born infants cared for in the neonatal units suffer from memory and learning deficits. Prematurity diminishes neurogenesis and synaptogenesis in the hippocampal dentate gyrus (DG). This dysmaturation of neurons is attributed to elevated PSD95, NMDR2A, and IGF1 levels. Since oestrogen treatment plays key roles in the development and plasticity of DG, we hypothesized that 17β‐estradiol (E2) treatment would ameliorate neurogenesis and synaptogenesis in the DG, reversing cognitive deficits in premature newborns. Additionally, E2‐induced recovery would be mediated by IGF1 signalling. These hypotheses were tested in a rabbit model of prematurity and nonmaternal care, in which premature kits were gavage‐fed and reared by laboratory personnel. We compared E2‐ and vehicle‐treated preterm kits for morphological, molecular, and behavioural parameters. We also treated kits with oestrogen degrader, RAD1901, and assessed IGF1 signalling. We found that E2 treatment increased the number of Tbr2^+^ and DCX^+^ neuronal progenitors and increased the density of glutamatergic synapses in the DG. E2 treatment restored PSD95 and NMDAR2A levels and cognitive function in preterm kits. Transcriptomic analyses showed that E2 treatment contributed to recovery by influencing interactions between *IGF1R* and neurodegenerative, as well as glutamatergic genes. ERα expression was reduced on completion of E2 treatment at D7, followed by D30 elevation. E2‐induced fluctuation in ERα levels was associated with a reciprocal elevation in IGF1/2 expression at D7 and reduction at D30. ERα degradation by RAD1901 treatment enhanced IGF1 levels, suggesting ERα inhibits IGF1 expression. E2 treatment alleviates the prematurity‐induced maldevelopment of DG and cognitive dysfunctions by regulating ERα and IGF1 levels.

## 1 INTRODUCTION

About 380,000 babies are born prematurely each year in the United States. Premature neonates are raised in a stressful environment of Neonatal Intensive Care Units (NICU), fed often on artificial formula and taken care by nurses in place of their biological mothers. Children born very premature (<1500 g birth weight) are at increased risk for learning disabilities and long‐term memory deficits. Prematurely‐born infants and adults display reduced volume of the dentate gyrus (DG) and overall hippocampal atrophy.^1^, ^2^ This can be attributed to premature cessation in the supply of oestrogen, other hormones and growth factors as well as to nonmaternal care of very premature infants in the stressful environment of NICUs.^3^ Oestrogen plays key roles in spinogenesis, synaptogenesis and the overall development of DG and hippocampus.^4^ We, therefore, asked whether oestrogen replacement would restore DG development and the cognitive deficits in prematurely delivered rabbit newborns and if so, what the underlying mechanism(s) would be.

MRI studies have shown that hippocampal volumes are reduced in prematurely‐born infants compared with their term‐born peers.^2^, ^5^, ^6^ Quantification of hippocampal subfields showed diminished volumes of DG, CA1‐4 and subiculum in preterm‐born adults at the age of 26 years; and evaluation of cognitive performance on them showed that the left DG volume mediated the effects of prematurity.^1^ Accordingly, 30%–60% of preterm‐born children suffer from cognitive and executive dysfunction, which impacts their school performance.^7^, ^8^ Magnetoencephalography has assessed the functional network underlying work memory, which shows a less mature pattern of function recruitment during working memory maintenance in preterm‐born children relative to term controls.^9^ Implicit with this notion, studies in a rodent model show that maternal separation increases anxiety‐related behaviour and changes spine density in CA1.^10^, ^11^ At the synaptic level, early neonatal stress accelerates the developmental shift in the ratio of NMDA receptor subunits, NR2A and NR2B, which leads to precocious upregulation of NR2A in the hippocampal CA1 region.^12^ In our recent study, we have demonstrated that prematurity reduces the maturation of synapses in the DG and elevates the expression of PSD95, NMDA receptor (NR)‐1, and NR‐2A.^13^ More importantly, insulin growth factor (IGF1) receptor inhibition reverses cognitive deficits, increases the density of glutamatergic, and rescues NR2B and PSD95 levels in the DG of premature kits.^13^ Hence, the abnormal development of the DG in premature infants contributes to cognitive deficits and is mediated by IGF1.

Oestrogen is a critical regulator of synaptic plasticity and activates signalling pathways in the CA1 hippocampus to promote both spinogenesis and synaptogenesis.^4^ It stimulates signal transduction pathways through oestrogen receptor (ER)‐α and ‐β. Oestrogen activates LIMK1 and cofilin phosphorylation to facilitate filopodia formation and increases PSD95 translation via phosphatidylinositol 3‐kinase (PI3K) activation of Akt.^4^ Similarly, IGFI activates IGF‐receptor to recruit phosphatidylinositol 3‐kinase (PI3K)/Akt pathway, thereby downregulating GSK3 in the aging brain.^14^, ^15^ Thus, there is a crosstalk between oestrogen receptor and IGF1 and both of them affect common second messenger signalling pathways, including PI3/AKT, MAP kinase, glycogen synthase kinase 3, and β‐catenin.^16^ In addition, oestrogen treatment offers neuroprotection in animal models of Alzheimer's disease, stroke, and neonatal hypoxia‐ischaemia.^16^, ^17^ However, the early and delayed effect of oestrogen treatment on DG development in the neonatal model of prematurity and nonmaternal care has not been studied.

We hypothesized that oestrogen treatment would restore abnormal maturation of DG and reverse cognitive deficits in prematurely‐born rabbits. Since IGF1 plays a crucial role in mediating dysmaturation of the DG and cognitive deficits in premature rabbits,^13^ we also postulated that the effect of oestrogen treatment is mediated by IGF1 signalling in premature kits. We tested these hypotheses in a model of prematurely delivered rabbits (E29, term = 32d) that were reared by laboratory personnel. The model reproduces the effect of premature birth and neonatal stress (nonmaternal care and formula feeding) suffered by the infants reared in the neonatal units.^3^, ^13^

## 2 MATERIALS AND METHODS

### 2.1 Experimental groups

The present study was reviewed and approved by the Institutional Animal Care and Use Committee of Albert Einstein College of Medicine, Bronx, NY. For this study, we purchased timed‐pregnant New Zealand white rabbits from Charles River Laboratories (Wilmington, MA). We performed Caesarean sections and delivered premature kits at embryonic day 29 (rabbit gestation 32 days). After the C‐section and delivery of preterm kits, we euthanized the mother doe. Preterm formula‐fed kits were brought to the laboratory immediately at birth, cared for in an infant incubator at 35°C and gavage‐fed through an orogastric tube by laboratory personnel. We employed rabbit milk replacer (Wombaroo) to gavage‐feed the kits an amount of 2–3 mL every 12 h (100 mL/kg/day) for the first 2 days, and feeds were gradually advanced to 125, 150, 200, 250 and 280 mL/kg at postnatal day (D) 3, D5, D7, D10 and D14, respectively. After D21, we introduced lettuce, cabbage, cauliflower, carrots and broccoli to gradually wean the kits to solid food. As soon as the kits started eating vegetables, gavage feeding was gradually terminated by D24 in preterm kits. Rabbit kits included both males and females. In order to confirm the sex of the kits, we performed RT‐qPCR on brain tissues using SRY TaqMan probes.

### 2.2 Oestrogen treatment

The preterm rabbit kits were treated daily with either intramuscular E2 (Sigma‐Aldrich) or vehicle for 7 days, starting at D1 until D7. The neurobehavioral tests were performed at 30 days of postnatal age, and then, they were euthanized. Oestrogen was administered in a dose of 200 μg/kg. DMSO was used as a vehicle.

### 2.3 Blocking ERα

We used RAD1901 to block ERα. Preterm rabbit kits were randomized into two treatment groups: oral RAD1901 or methylcellulose (vehicle). RAD1901 (MedChemExpress) was dissolved in methylcellulose and was gavage‐fed to rabbit kits in a dose of 10 mg/kg once a day for 7 days, starting at D1.

### 2.4 Neurobehavioral assessment

Oestrogen‐ and vehicle‐treated rabbit kits were examined for their neurobehavioral performance at D30 as before. Any‐Maze Software (Stoelting Company) was utilized to collect and analyse all behavioural assessments by tracking their movement/behaviour and time spent in delineated zones, using an overhead video camera. The *open field* test was employed for general activity levels, gross locomotor activity, speed and time spent in the centre and periphery of the arena. Animals were positioned in a square arena (40 × 40 × 20 inches) and allowed to move spontaneously for 10 min. Distance moved, velocity and time spent in delineated zones were noted. *Novel Object Recognition* assessed cognition and recognition memory by the spontaneous tendency to spend more time exploring a novel object relative to a familiar one. The kit was put in a small arena (40 × 40 × 30 inches) with 2 identical objects for 3 min, followed by 1 h rest in a home cage. This was repeated 3 times so that kits got used to both objects. The next day, the kits were placed in the arena for 3 min with one of the known objects replaced with a novel object in the same location. We computed the duration the rabbit spent with the novel versus the known object. *The* o*bject placement test* was used to evaluate *s*patial memory in a similar manner as the novel object test, except that in trial 2 the kit was exposed again to the same 2 identical objects; of these one was displaced in space. *Elevated Plus Maze* was performed to assess anxiety‐related behaviour. The maze consists of 4 arms that are 30 inches above the ground. Two arms had 20‐inch‐high opaque sides. Kits were put in the centre of the maze and allowed to explore for 5 min. The preference for open arms over closed arms was a measure that reflected anxiety‐like behaviour. *The modified Barnes Maze test evaluated the memory and learning of the kits*. The test was done on a Barnes maze apparatus, which has 20 indentations around the perimeter of a circular arena. The apparatus was placed in the centre of a small room with unique symbols on each of the four walls. One of the indentations contained a food pellet, which the rabbit was expected to find. The kits were starved overnight to augment the motivation for seeking the treat. The kits were placed in the centre of the arena and given 2 min to search for the food. If the rabbit failed to find the food after 2 min, the investigator showed the kit the appropriate indentation, and then, the kit was returned to the home cage for rest. This training has repeated a total of 4 times, terminated the trial after the consumption of the food. The next day, the rabbit was again placed in the arena for 90 sec to fi the food. Spatial memory was measured based on the amount of time the rabbit spent searching the indentations carrying food.

Immunohistochemistry (IHC) and stereological quantifications, western blot analyses and quantitative real‐time polymerase chain reactions are described in Appendix S1.

Isolation of Nuclei and FACS sorting of hippocampal dentate gyrus granule cells and RNAseq are also detailed in Appendix S1.

### 2.5 Statistical analyses

Data are expressed as means ± SEM. All the behavioural tests were analysed by *t*‐test. The number of NeuN^+^, GABA^+^, Prox1^+^ and calbindin^+^ cells in the DG at D14 and D30 were evaluated by two‐way anova. All post hoc comparisons to test for differences between means were done using the Tukey multiple comparison tests at the 0.05 significance level. Dendritic length, branching, and density as well as western blot analysis data were compared between full‐term and preterm groups by the unpaired *t*‐test. Statistical significance was set using an α level of 0.05.

## 3 RESULTS

### 3.1 E2 treatment enhances neurogenesis in the dentate gyrus

**FIGURE 1:**
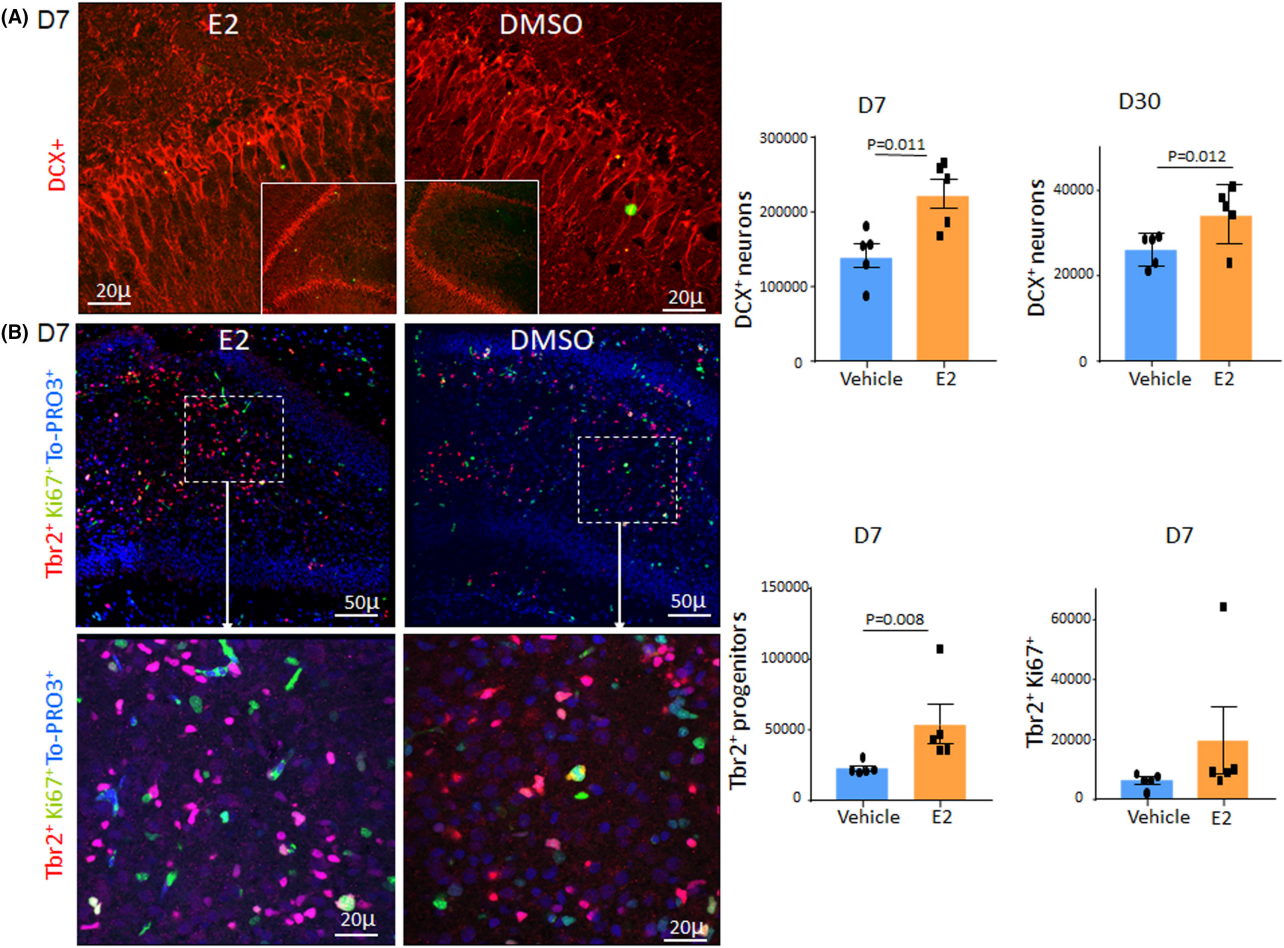
E2 treatment enhances neurogenesis in the DG. (A) Representative immunofluorescence of the coronal sections from the DG of E2‐ and vehicle‐treated rabbits at D7 labelled with DCX antibody. Note DCX^+^ migratory neurons are more abundant in E2‐treated group compared with control at D7. Bar graphs are mean ± SEM, *n* = 5 per group, Student's *t*‐test used. (B) Representative immunofluorescence of the coronal sections from the DG of E2‐ and vehicle‐treated kits at D7 labelled with Tbr2 and Ki67 specific antibodies. Lower panel is a high magnification image of the boxed area in the upper panel. Note Tbr2 progenitors are more abundant in E2‐treated group compared with control. Scale bar, 50 μm (upper panel), 20 μm (lower panel). Bar graphs are mean ± SEM, *n* = 5 per group, Student's *t*‐test used.

The dentate granule cell layer in rodents has the unique property of lasting neurogenesis.^18^ Our previous study revealed that premature birth reduces neurogenesis and enhances the maturation of neurons in the DG.^13^ Since E2 enhances neurogenesis in the DG of adult animals,^19^ we postulated that E2 treatment would enhance neurogenesis in premature rabbits. To this end, we treated preterm infants (E29) with 17β‐estradiol (E2) for 7 days starting at postnatal day (D)1, and we assessed neuronal production in the DG at D7 and D30. We labelled coronal sections with doublecortin (DCX, migratory neurons). We found that E2 treatment increased the number of DCX^+^ neurons in E2‐treated kits compared with vehicle controls at both D7 and D30 (*p* = 0.011 and 0.012, Figure 1A). We next evaluated Tbr2^+^ glutamatergic neuronal progenitors and found that the number of total Tbr2^+^ cells was higher in number in E2‐treated kits relative to controls at D7 (*p* = 0.008, Figure 1B). The cycling Tbr2^+^ cells showed an insignificant trend toward an increase in the subgranular layer of the DG of E2‐treated preterm kits relative to vehicle controls (*p* = 0.095, Figure 1B). Tbr2^+^ cells were few in the DG at D30 and thus were not quantified. Together, the data suggest that E2 treatment restores neurogenesis in preterm kits.

### 3.2 Oestrogen treatment reduces the number of interneurons in the dentate gyrus

**FIGURE 2:**
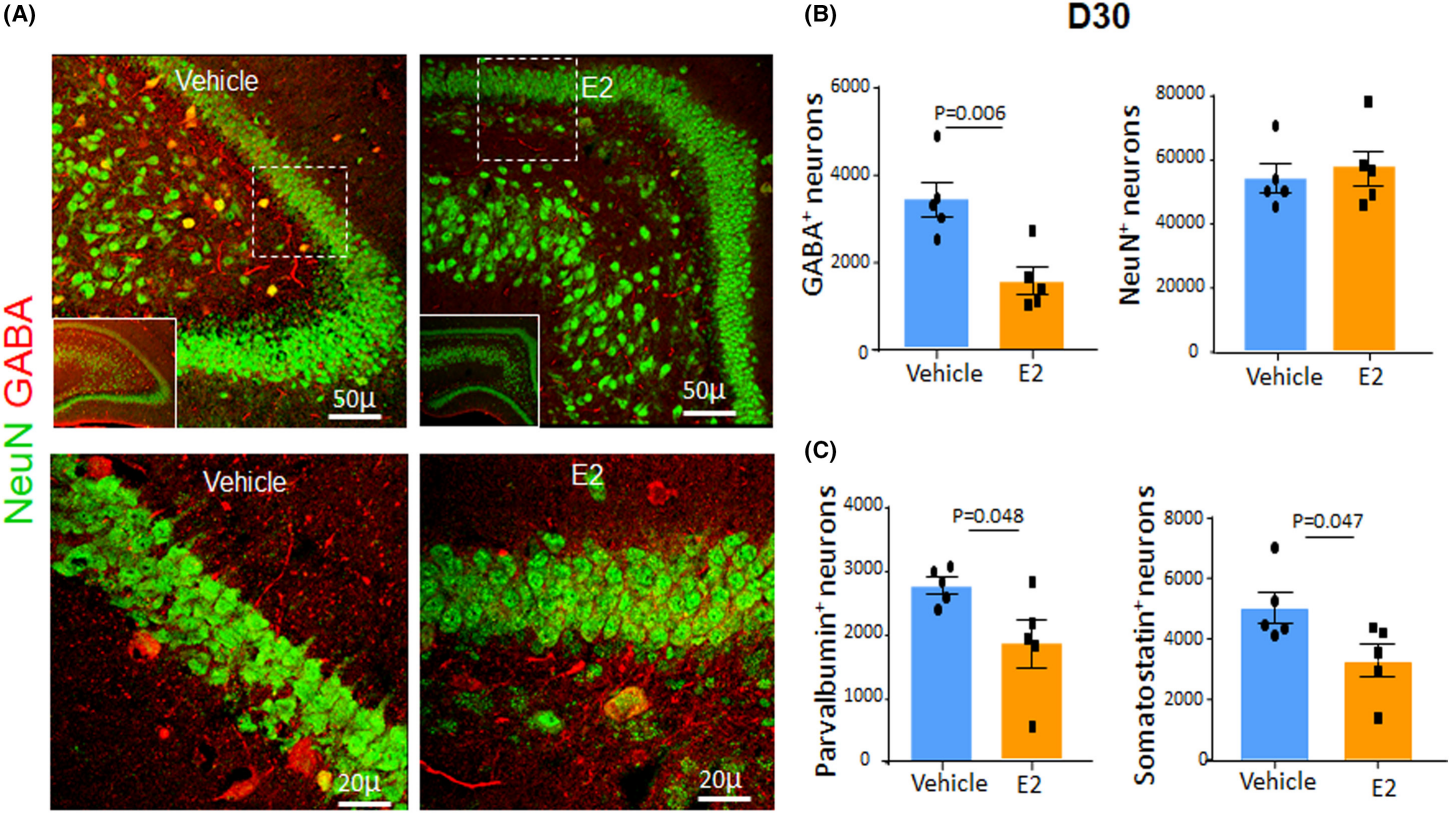
E2 treatment reduces the number of interneurons in the DG. (A) Representative immunofluorescence of the coronal sections from the DG of E2‐ and vehicle‐treated rabbits at D30 labelled with NeuN (green) and GABA (red) specific antibodies. Lower panel is a high magnification image of the boxed area in the upper panel. Bar graphs are mean ± SEM, *n* = 5 per group. Stereological quantification shows that number of GABA^+^ cells is less in E2‐treated kits compared with control. Inset shows a low power view of the DG. (B) Data in are mean ± SEM, *n* = 5 each group, Student's *t*‐test used. Both PV+ and SST+ neurons were less in number in E2‐treated kits compared with the vehicle controls.

Our previous study has shown that the number of NeuN^+^ and GABA^+^ neurons is higher in preterm kits (E29) relative to term controls (D30), suggesting prematurity‐induced accelerated maturation of neurons in the DG. Hence, we sought to assess the effect of E2 on the NeuN^+^ and GABA^+^ neuronal populations in the DG. Our stereological quantification showed that E2 treatment reduced the number of GABA^+^ interneurons in the DG relative to vehicle controls at D30 (*p* = 0.006) but not NeuN+ neurons (Figure 2A,B). Western blot analyses showed that E2 treatment did not significantly affect NeuN and GAD67 (glutamate decarboxylase in GABA^+^ neurons) protein levels relative to vehicle controls at D30 (Figure S1A). Consistent with the reduction in the number of GABA^+^ interneurons, PV^+^ and SST^+^ interneurons were also reduced in E2‐treated kits compared with vehicle controls at D30 (*p* = 0.046 and 0.047, respectively, Figure 2C). GABA, PV and SST immunoreactivity was weak‐to‐absent in D7 kits and thus were not evaluated.

Interneuron subtypes display diversity in morphology and function. Our previous studies have shown a prematurity‐induced reduction in the number of calbindin^+^ interneurons in the DG.^13^ Hence, we quantified calbindin interneurons as well as Prox1^+^ granule neurons in E2‐treated kits relative to vehicle controls. Prox1, a prospero‐related homeobox gene, is a key regulator of granule cell maturation and is essential for the formation of learning and memory.^20^ We found that E2 treatment increased the number of both Prox1^+^ and calbindin^+^ neurons in the dentate gyrus (*p* = 0.02 and 0.016, respectively. Figure S1B), thereby restoring the prematurity‐induced deficit in calbindin population. Together, the data suggest that E2 treatment reverses the prematurity‐induced elevation in the number of GABA^+^ interneurons and reduction in the number of calbindin^+^ neurons, thereby contributing to restoring the maturation of DG in preterm kits.

### 3.3 E2 treatment enhances synaptogenesis

**FIGURE 3:**
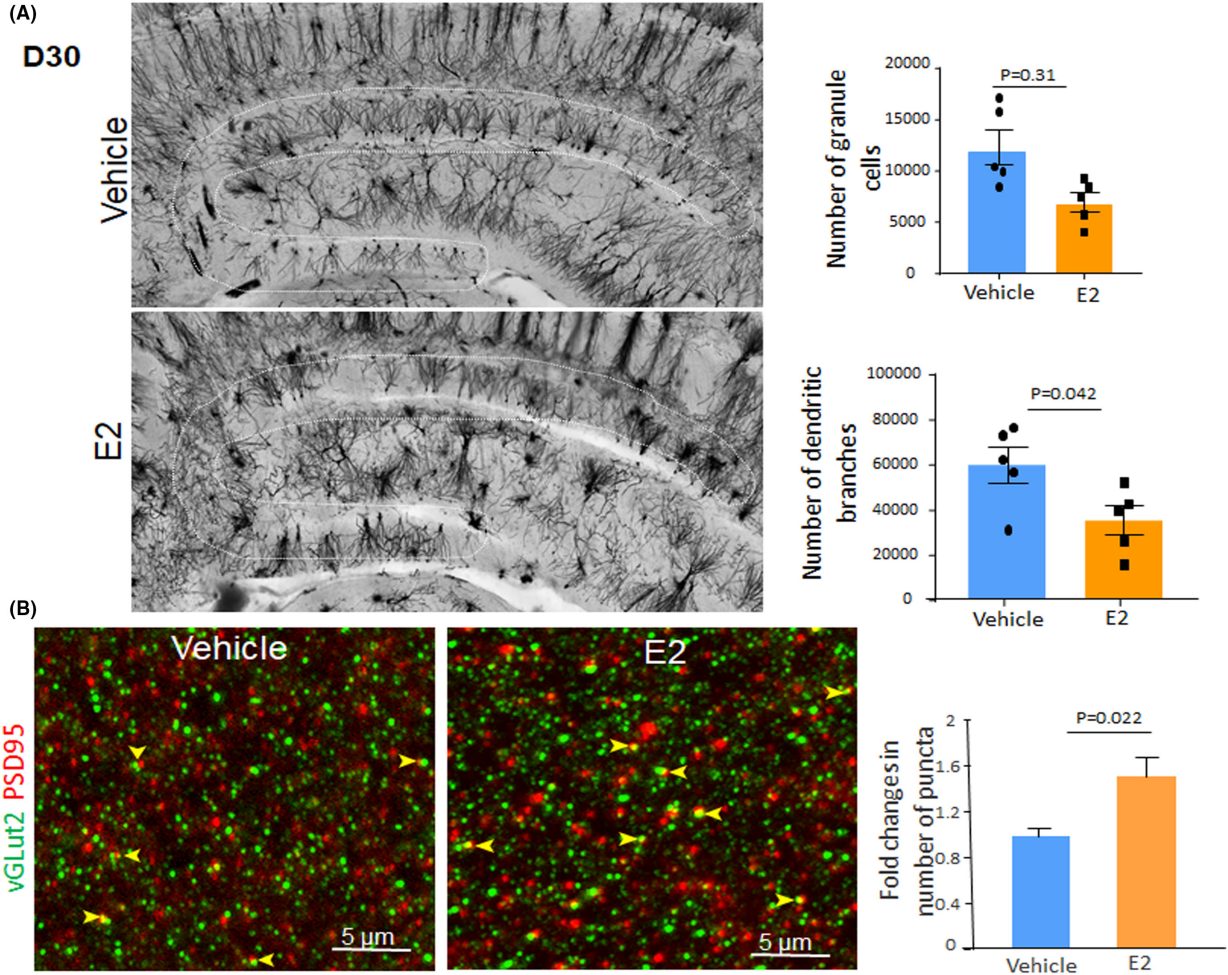
E2 treatment enhances synaptogenesis. (A) Representative images of Golgi‐stained sections from DG of E2‐treated kits and vehicle controls. The demarcated region (broken lines) indicates supra‐ and infrapyramidal blade. Data are means ± SEM (*n* = 5 each), Student's *t*‐test used. The number of granule cells and the dendritic branching were lower in E2‐treated kits relative to controls. (B) Representative images taken from the coronal section of the DG of D30 vehicle‐ and E2‐treated kits, double‐labelled with VGlut2 and PSD95 antibodies. The colocalization of VGlut2 and PSD95 (arrowheads) indicates a glutamatergic synapse (arrowhead). Bar charts show means ± SEM, *n* = 5 each group, Student's *t*‐test used. The density of synaptic puncta was higher in E2‐treated kits relative to controls.

Prematurity‐associated stress increases the number of granule cells, reduces the density of the mature dendritic spine and synapses in DG, and is associated with cognitive deficits.^13^ Therefore, we evaluated the effect of E2 on dendritic branching and dendritic spines in the supra‐ and infrapyramidal blades of the DG in Golgi‐stained coronal slices. We found that granule cells were diminished in E2‐treated kits compared with vehicle controls (*p* = 0.031, Figure 3A). Similarly, the number of all dendritic branches was also few in E2‐treated preterm kits relative to vehicle controls (*p* = 0.042, Figure 3A). However, the number of dendritic branches per granule cell (ratio of the total number of branches and the total number of cells) was greater in E2‐treated kits relative to controls. Despite a reduction in the absolute number of granule cells, the volume of DG was increased in E2‐ relative to vehicle‐treated kits at D30 (0.03582 ± 0.0022 vs.0.0428 ± 0.0012 mm^3^, *p* = 0.014). The increase in the volume of DG is attributed to an increase in neuropil number, synapses, and extracellular matrix in E2‐treated kits compared with vehicle controls.^21^, ^22^ The E2‐induced reduction in the number of granule cells in Golgi‐stained sections, however, was not associated with a similar reduction in the number of NeuN^+^ neurons demonstrated in immune‐stained sections of the DJ.

We next stained glutamatergic synapses in the coronal section from E2‐ and vehicle‐treated preterm kits using presynaptic markers, VGlut2, and postsynaptic density protein 95 (PSD95) antibodies. The co–localization of these markers indicated glutamatergic synapses (Figure 3B). Quantification showed that the density of synaptic puncta in the molecular layer (perforant path‐granule cell synapse) was higher in E2‐treated preterm kits compared with vehicle controls at D30 (*p* = 0.022). Together, these studies reinforce the notion that E2 treatment ameliorates dysmaturation of DG by enhancing synaptogenesis.

**FIGURE 4:**
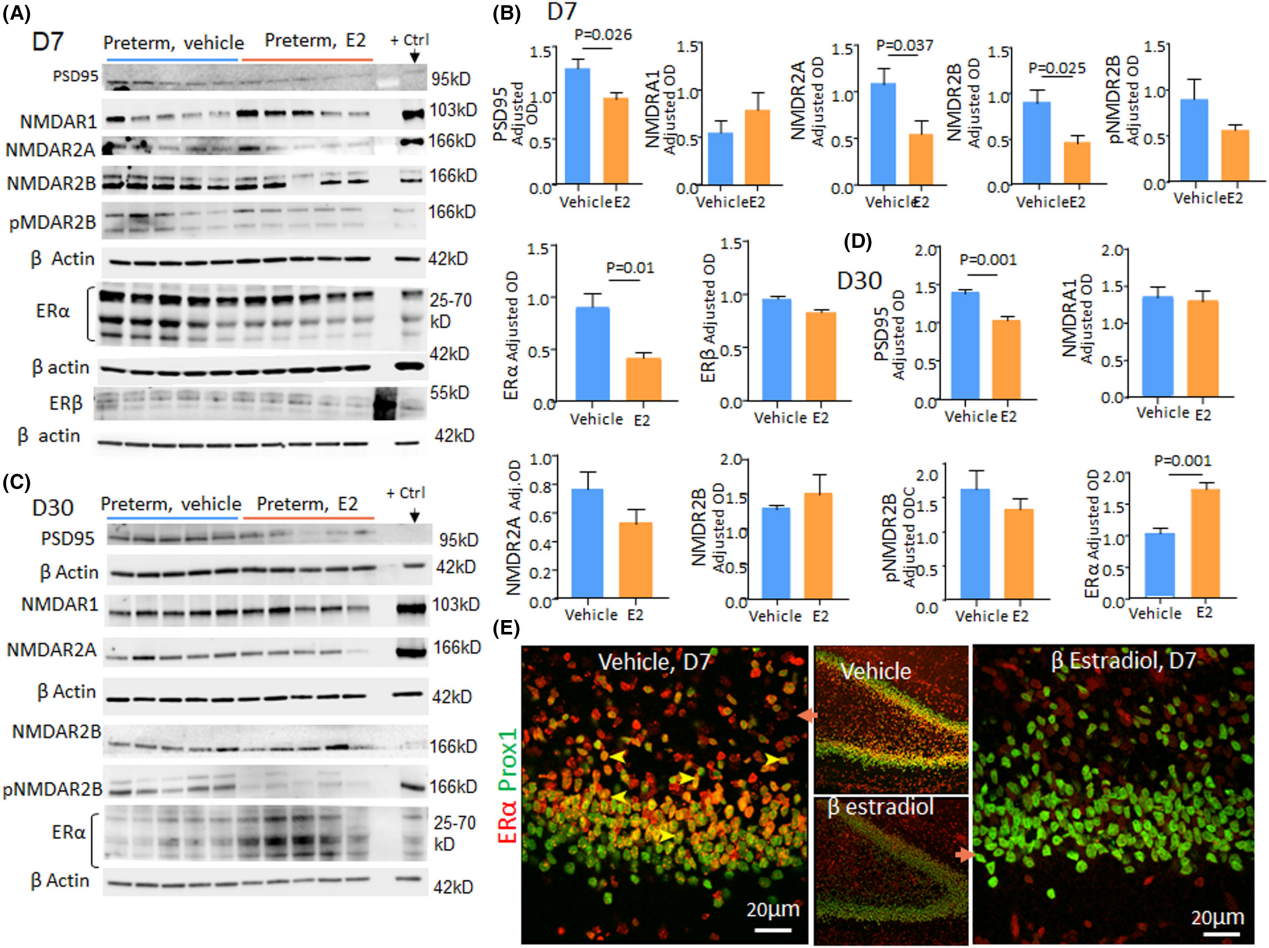
E2 Treatment modulates NMDA receptor subtypes and oestrogen receptor α in premature kits. (A, B) Typical western blot analyses on homogenates from preterm kits at D7 using PSD95, NMDAR1, NMDAR2A, NMDAR2B pNMDAR2B and ERα antibodies. Bar charts show means ± SEM, *n* = 5 each group, Student's *t*‐test used. Mouse brain was used as positive control. Protein concentrations were normalized to ß‐actin. Note reduced levels of PDS95, NMDAR2A, NMDAR2B and ERα E2‐treated kits relative to controls at D7. (C, D) Representative western blot analyses for PSD95, NMDAR1, NMDAR2A, NMDAR2B pNMDAR2B and ERα in E2‐ and vehicle‐treated kits at D30. Bar charts show means ± SEM, *n* = 5 each group, Student's *t*‐test used. Protein concentration for all markers was normalized to ß‐actin. PDS9 is reduced and ERα was higher in E2‐treated kits compared with controls at D30. (E) Coronal sections from DG were double labelled with ERα and Prox1 antibodies at D7. Arrowhead indicates the colocalization of ERα and Prox1. Note reduced ERα expression in E2‐treated kits compared with abundant ERα on Prox1+ granule cells in vehicle controls.

PSD95 and NMDA receptor (NMDAR) play key roles in dendritic spine morphology and function.^23^ Our previous studies show that premature kits exhibit elevation in PSD95, NMDAR1, and NMDAR2A receptor levels, which likely reduces the formation of glutamatergic synapses.^13^ Hence, we reasoned that E2 treatment might reverse the elevation in PSD95 and subunits of NMDAR protein levels in preterm kits. Western blot analyses showed that PSD95 levels were reduced in E2‐treated preterm kits relative to vehicle controls at both D7 and D30 (*p* = 0.026 and 0.001, respectively, Figure 4A–D). Similarly, protein levels of NMDAR2A and NMDAR2B were significantly reduced in E2‐treated kits relative to vehicle controls at D7 (*p* = 0.037, 0.025), a difference not observed for NMDAR1 and phospho‐NMDAR2B. The levels of NMDAR1, NMDAR2A, NMDAR2B, and phospho‐NMDAR2B were comparable between the two groups at D30. Together E2‐induced reduction in PSD95, NMDAR2A, and NMDAR2B levels likely enhanced the maturation of dendritic and glutamatergic synapses in preterm kits.^24^

### 3.4 E2 treatment reduces oestrogen receptor α in premature kits

E2 treatment mediates synaptic plasticity in the hippocampus by oestrogen receptors, ERα, ERβ, and GPR30. Selective activation of ERα increases synaptic plasticity and cognitive performance in rodents. Hence, we assessed the effect of E2 treatment on the expression of ERα and ERβ in the DG.^25^

Western blot analyses showed that ERα (ERα, 25–70 kD) was reduced at D7 (*p* = 0.01, Figure 4A,B) and elevated at D30 (*p* = 0.0012, Figure 4C,D) in E2‐treated kits relative to controls. However, ERβ levels were comparable at D7. Consistent with western blot analyses, immunostaining showed that ERα expression was reduced at D7 (Figure 4E) and elevated at D30 in E2‐treated kits (image not shown). Hence, E2 treatment induces a biphasic change in ERα expression resulting in early downregulation and later upregulation in its levels.

### 3.5 E2 treatment reverses cognitive dysfunction in premature kits

**FIGURE 5:**
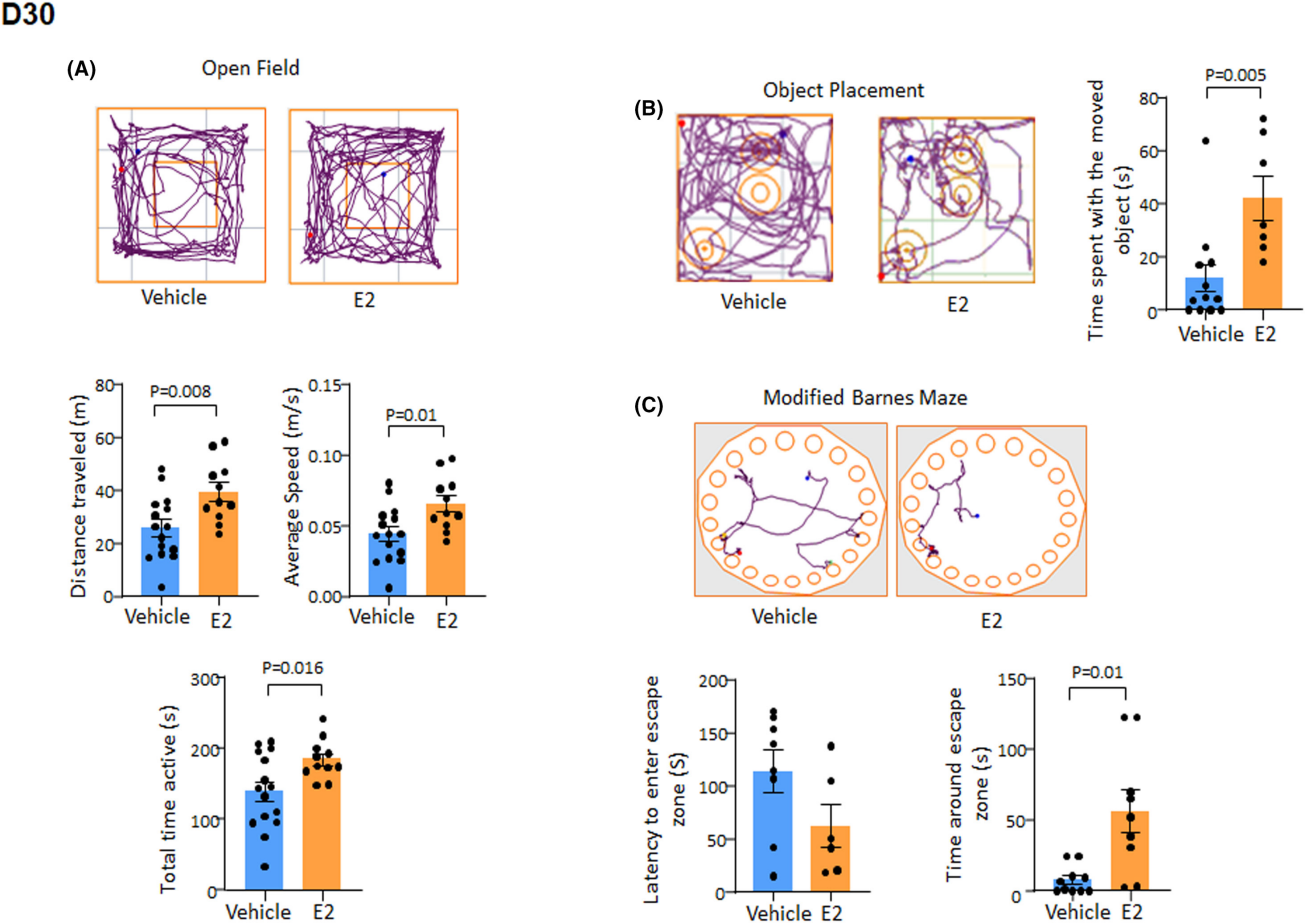
E2 treatment reverses cognitive dysfunction in premature kits. (A) Representative track plots of E2‐ and vehicle‐treated kits in the open field test at D30. Bar charts show mean ± SEM, *n* = 11–15 each group, Student's *t*‐test used. Note that distance travelled, average speed and total time active were higher in E2‐treated kits relative to controls. (B) Representative track plots of E2‐treated kits compared with control kits in the object placement test at D30. Bar charts show mean ± SEM (*n* = 10–15 each group), Student's *t*‐test used. Note that E2‐treated kits spent more time with moved object compared with control. (C) Typical track plots of E2‐treated kits compared with controls in modified Barnes maze test at D30. Bar charts show mean ± SEM (*n* = 8–10 each group). Note latency to find the escape zone was shorter and time spent around the escape zone was longer for E2‐treated kits relative to vehicle control. Student's *t*‐test used.

Prematurity reduces spatial recognition and memory in rabbit kits. Since E2 treatment enhances synaptogenesis, we postulated that E2 treatment might enhance cognitive functions. To this end, we performed open field, object placement and modified Barnes maze tests for E2‐ and vehicle‐treated kits at D30 (Figure 5A,D,C).

The open field test showed that the total distance travelled, average speed and total time active were greater in E2‐treated kits compared with vehicle controls (*p* = 0.008, 0.01 and 0.016, respectively, Figure 5A). The object placement test revealed that the time spent with the moved object, indicative of long‐term memory of the previous location, was longer in E2‐treated preterm kits compared with vehicle controls (*p* = 0.005, Figure 5B). We next carried out a modified Barnes maze test using a circular arena with 20 indentations around the perimeter.^3^ Rabbit kits were starved overnight and were allowed to search for food hidden in one of the indentations (the escape zone). The time taken to find the escape zone was similar between E2 and vehicle treated kits during three training sessions (*p* = 0.1). However, the following day, time spent around the escape zone was longer for E2‐treated kits relative to vehicle controls (*p* = 0.01). Accordingly, the latency to find the escape zone was shorter in E2‐treated kits (*p* = 0.0031, Figure 5C). These data indicate that E2 treatment improved spatial recognition and long‐term memory in preterm kits. Together, E2 treatment enhances the motor as well as recognition and spatial memory of preterm kits.

### 3.6 Rise in IGF1 level after completion of E2 treatment at D7 and a subsequent decline at D30

IGF1 is a principal growth factor that promotes neurogenesis, synaptogenesis and development of DG and could contribute to neurobehavioral deficits.^26^, ^27^, ^28^ Moreover, E2 and IGF1 appear to have synergistic effects.^15^ We thus evaluated the effect of E2 on IGF1 expression in the DG of premature kits at D7 and D30.

**FIGURE 6:**
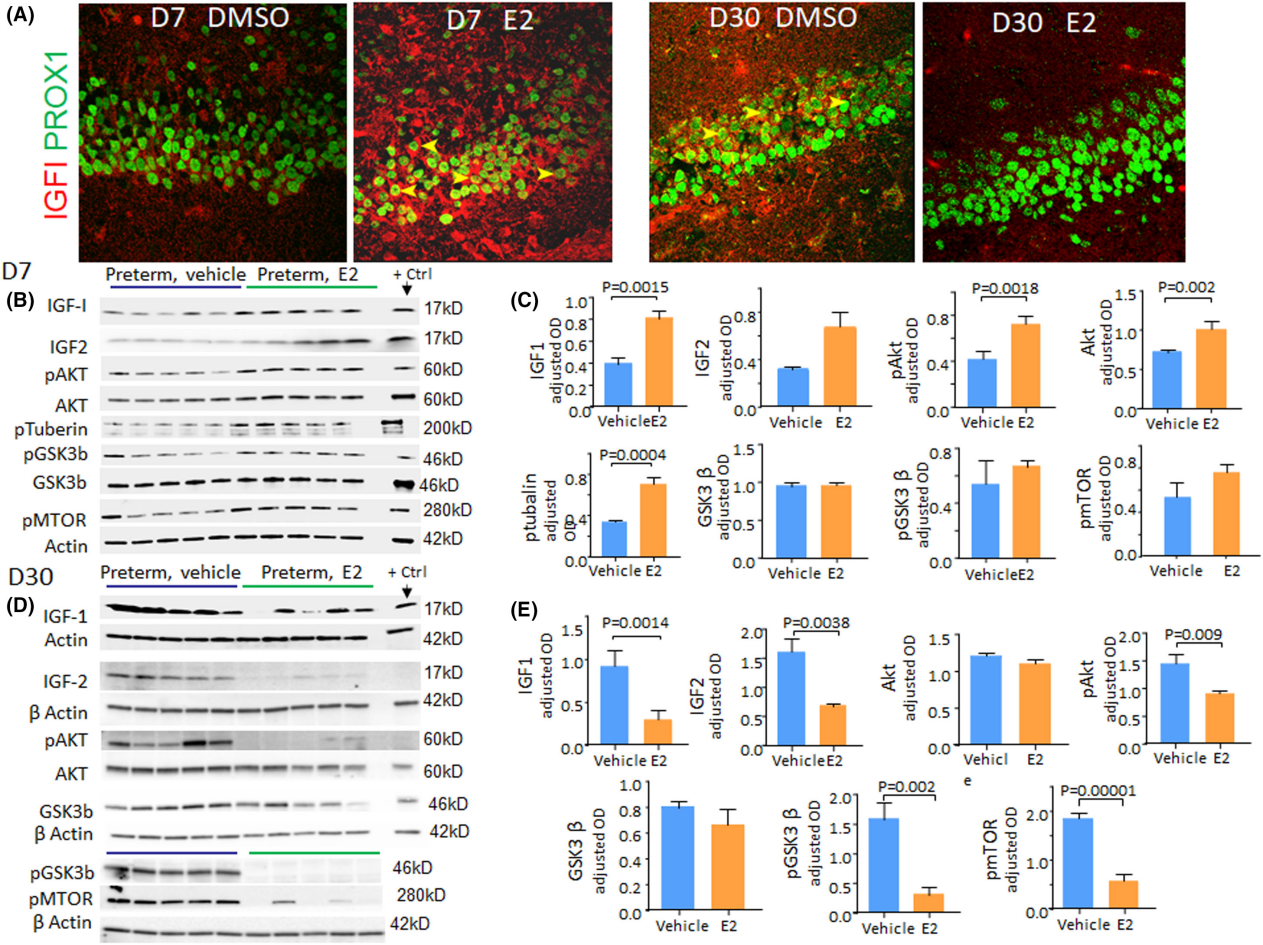
E2 Treatment induces biphasic changes in IGF1 levels and its downstream signalling. (A) Representative immunofluorescence of cryosections from D7 and D30 kits labelled with Prox1 and IGF1 specific antibodies. IGF1 expression in the DG (arrowhead) was higher in E2‐treated kits at D7 but reduced in E2‐treated kits at D30 compared with vehicle controls. (B) Representative western blot analyses for IGF1, IGF2, pAKt, AKT, GSK3β, pGSK3 and pMTOR protein homogenates of vehicle‐ and E2‐treated preterm kits at D7. Bar charts show means ± SEM, *n* = 5 each group, Student's *t*‐test used. Values were normalized to ß‐actin. Note elevated IGF1, IGF2, pAKT, AKT and p‐tubulin protein levels in E2‐treated kits compared with controls at D7. (C) Representative western blot analysis for IGF1, IGF2, pAKt, AKT, GSK3β, pGSK3 and pMTOR protein in homogenates from vehicle and E2‐treated preterm kits at D30. Mouse brain was used as a positive control. Values were normalized to ß‐actin. Bar charts show means ± SEM, *n* = 5 each group, Student's *t*‐test used. Note IGF1, IGF2, pAKt, pGSK3 and pMTOR protein levels were lower in E2‐treated kits compared with controls at D30.

Immunohistochemistry showed the immunoreactivity to IGF1 is greater in E2‐treated kits compared with controls at D7 (Figure 6A). Consistent with immunostaining, western blot analyses confirmed that IGF1 levels were higher in E2‐treated kits relative to controls at D7 (*p* = 0.0015, Figure 6B,C). In agreement with IGF1 levels, the downstream molecules Akt, pAkt and p‐tubulin levels were elevated in E2‐treated kits compared with vehicle controls at D7 (*p* = 0.0018, 0.002 and 0.0004, respectively, Figure 6B). However, GSK3β, p‐GSK3β and pmTOR levels were comparable between the groups at D7. We next quantified IGF1 and its downstream signalling molecules at D30. In contrast to D7, IGF1 levels were significantly reduced in E2‐treated kits compared with controls at D30 (*p* = 0.014, Figure 6D,E). Accordingly. pAkt, p‐GSK3β and pmTOR were also reduced in E2‐treated kits relative to vehicle controls at D30 (*p* = 0.009, 0.002, 0.0001). However, total Akt and GSK3β levels were comparable between the two groups. Together, E2 treatment results in an initial increase in IGF1 levels, which declines significantly below the control levels at D30.

### 3.7 Degradation of oestrogen receptor α elevates IGF1 levels

**FIGURE 7:**
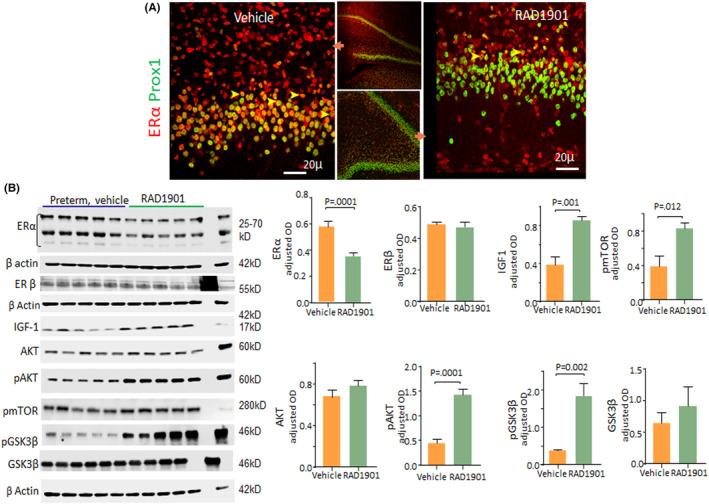
Degradation of Oestrogen receptor α elevates IGF1 signalling: (A) Cryosections from DG of RAD1901‐ and vehicle‐treated kits were labelled for ERα and Prox1 antibodies. Representative image shows that the RAD1 treatment reduces ERα expression (arrowhead) in the DG compared with vehicle controls. (B) Representative western blots for Erα, Erβ, IGF1 pMTOR, pERα, pGSK3b, GSK3b, pTuberin, pAKT and AKT protein in homogenates of vehicle and RAD1901‐treated kits at D7. Values were normalized to ß‐actin. Bar charts show means ± SEM, *n* = 5 each group, Student's *t*‐test used. Note that RAD1901 treatment reduced ER α levels. Also note elevated IGF1, pMTOR, pAKt/Akt, GSK3 β protein levels in RAD1901‐treated kits relative to control kits at D30.

Since E2 treatment reduced ERα and was associated with the upregulation of IGF1 levels and activation of downstream molecules, we postulated that diminution in ERα triggers IGF1 signalling. To this end, we treated E29 rabbit kits with Elacestrant (RAD1901) or vehicle, starting from D1. Immunohistochemical labelling showed that the expression of ERα was reduced in the DG in RAD1901‐treated kits compared with controls at D7 (Figure 7A).

Western blot analyses showed that ERα expression was reduced in the DG of RAD1901‐treated kits compared with controls (*p* = 0.001, Figure 7B), but a similar difference was not observed for ERβ. In addition, RAD1901 treatment increased IGF1 levels (*p* = 0.001) and its downstream molecules, including pmTOR, pAkt and p‐GSK3β (*p* = 0.012, 0.001 and 0.001, Figure 7B). In conclusion, the data suggest that degradation of ERα triggers upregulation of IGF1 and its downstream signalling.

### 3.8 Potential ERα sequences that regulate IGF1

To understand how E2 regulates IGF1, we performed a literature search and analysed two ChIP‐seq datasets, one from a human MCF7 cell line and the other from a mouse mammary gland.^29^, ^30^ We downloaded peak files of ChIP‐seq from GEO (GSE130032) and ArrayExpress (E‐MTAB‐158 and E‐TABM‐828) and uploaded in UCSC genome browser to check correlation with IGF1. We found two ERα receptor ChIP‐seq peaks in the human cell line, one at 2kb upstream of the transcription start site for IGF1 and the other in the intron region of IGF1 (likely in the enhancer region, Figure S2A). The analyses of mouse mammary gland data revealed three ERα receptor Chip‐seq peaks at about 40–60 kb upstream of the transcription start site for IGF1 (Figure S2B). We next performed Rabbit genome‐wide motif scan for ERα against rabbit genome using the HOMER (v4.11) module ‘scan Motif Genome‐Wide’ and found an ERα binding site at 20kb upstream of the transcriptional starting site of IGF1 (Figure S2C). This explains how ER‐α likely regulates IGF1. The results of our analyses in three species—human, mice and rabbit—are consistent with other studies^31^

### 3.9 E2 treatment induces transcriptomic changes in the dominion of granule cell proliferation, maturation and survival

To understand the mechanism, by which E2 treatment enhances cognitive function, we chose to perform transcriptomic studies in parallel with neurobehavioral function at D30. We isolated NeuN^+^Prox1^+^ cells from the DG, performed RNAseq and analysed granule cell transcriptome. We determined the transcriptomic regulation as (a) expression ratio /fold changes [X, negative for downregulation] and (b) weighted individual regulations [WIR].^32^ Table S1‐1 presents the significantly up‐regulated and Table S1‐2 the significantly downregulated genes together with their weighted contribution (WIR)^33^ in E2‐ vs. vehicle‐treated kits. The expressions of 10,451 genes were adequately quantified in all samples, 8645 of them encoding known proteins. Of these, the expression of 83 genes with known protein products was significantly elevated, and 37 genes were reduced in E2‐treated kits relative to vehicle controls. Our analyses showed that genes for cell cycle, neuronal differentiation, inflammation, apoptosis, purine metabolism and HIPPO signalling pathway were significantly altered. Gene‐promoting cell cycle including *CCNB1* (cyclin B1), CNNM2 (cyclin and CBS domain divalent metal cation transport mediator 2) and *KIF9* (kinesin family member 9) were reduced, whereas transcripts inhibiting cell cycle KIAA1456, KIAA1462 and LATS2 (large tumour suppressor kinase 2) were elevated. Genes‐promoting interneuron neurogenesis (like *DLX1*, distal‐less homeobox 1) was reduced, while genes enhancing interneuron maturation (such as *SOX6*, SRY‐box transcription factor 6) were elevated. The Hippo‐signalling pathway is a highly conserved and familiar tissue growth regulator, primarily dealing with cell survival, cell proliferation and apoptosis. Oestrogen treatment increases *TEAD1* (TEA domain transcription factor 1), *WNT2B* (Wnt family member 2B), *LATS2* (large tumour suppressor kinase 2) and *TP53BP2* (tumour protein p53 binding protein 2). Genes having a role in purine metabolism, *ADCY8* (adenylate cyclase 8) *and PDE5A/8A* (phosphodiesterase 5A/8A) were elevated, but *ENTPD3* (ectonucleoside triphosphate diphosphohydrolase 3) was reduced. Genes regulating apoptosis and inflammation, *CAPN5* (calpain 5) and *CAPS1* (calcyphosine) were increased; however, *C1QTNF2* (C1q and tumour necrosis factor‐related protein 2) and *CARD10* (caspase recruitment domain family member 10) were reduced. Gene‐regulating extracellular matrix and collagen synthesis, including *COL21A1*, *COL27A1*, *COL5A3*, were elevated. Oestrogen also affected lipid biosynthetic pathways modulating several genes including *KDSR* (3‐ketodihydrosphingosine reductase), *HTR2C* (5‐hydroxytryptamine receptor 2C), *GPAM* (glycerol‐3‐phosphate acyltransferase, mitochondrial), *INPPL1* (inositol polyphosphate phosphatase like 1), *PIGM* (phosphatidylinositol glycan anchor biosynthesis class M), *and LOC100343510* (isopentenyl‐diphosphate Delta‐isomerase 1). Interestingly, *TTN* (titin) had the largest positive contribution to the overall transcriptomic alteration (WIR = 4.85) and *ABI2* (abi interactor 2) the largest negative contribution (WIR = −2.16). High expression of TTN has been reported in gliomas,^33^ while ABI2‐deficient mice exhibit difficulties in learning and memory.^34^

### 3.10 E2 treatment ameliorates the interaction between *IGF1R* , neurodegenerative and glutamatergic genes

Since E2 mediates neuroprotective effects through IGF1‐IGFR signalling, we evaluated synergistic and antagonistic interactions between *IGF1R* genes and genes for neurodegeneration implicated in amyotrophic lateral sclerosis and Alzheimer's, Huntington's, Parkinson's and prion diseases. Based on the Principle of Transcriptomic Stoichiometry,^35^ requiring the coordinated expression of genes networked in functional pathways, we performed the pair‐wise product‐momentum Pearson correlation analysis between the (log_2_) expression levels of *IGF1R*. Seventy‐seven genes related to neurodegenerative diseases were quantified across biological replicas in the E2‐ and vehicle‐treated samples. Positive correlation meant the fluctuation of two genes in the same direction (synergistically pairing), whereas negative correlation implied the fluctuation of two genes in opposite directions (antagonistically pairing). A close to zero correlation meant a change in the expression of one gene had no impact on the other gene. In our analysis, synergic and antagonistic correlations were considered significant if the absolute value of the correlation coefficient exceeded 0.095. E2 treatment led to a statistically significant antagonism of *IGF1R* with the subunits *ATP5A1*, *ATP5G1*, *and ATP5O* of the ATP synthases H+ transporting complexes and reduced the synergistic coupling with the Parkinson's disease *SNCAIP* (synuclein alpha interacting protein; Figure S3A).

We next evaluated the effect of E2 treatment on the interaction between *IGF1R* and glutamatergic genes. The correlation analysis (Figure S3B) indicated that in E2‐treated animals the significant synergistic and antagonistic correlation of *IGF1R* with the 71 quantified glutamatergic synapse genes dropped from 38.81% (22.39% + 16.42%) to 7.46% (no antagonistic coupling), while the percentage of the independently expressed pairs increased from 1.49% to 5.97%. We speculate that E2 treatment enhances neurobehavioral function by reducing the effect of IGF1R on glutamatergic synaptic transmission and antagonizing genes for the neurogenerative disease.

## 4 DISCUSSION

Prematurity‐associated cognitive deficits and developmental delays are global health problems. About 11% of infants are born premature, and prematurely‐born children and adolescents often suffer from learning disabilities and long‐term memory deficits. Premature birth terminates the exposure of infants to placental and maternal oestrogen and thus, oestrogen supplementation for premature newborns would be physiological.^36^ Herein, we showed that E2 treatment in premature kits restored neurogenesis, reversed dysmaturation of granule cells and promoted synaptogenesis, thereby improving their cognitive function. In addition, ERα expression was reduced at the termination of E2 treatment at D7, followed by a rebound rise at D30. E2‐induced fluctuation in ERα levels was linked with a reciprocal increase in IGF1/2 expression at D7 and reduction at D30. The study highlights the oestrogen‐led recovery of prematurity‐induced disruption in DG development, which is mediated by ERα and IGF1 signalling.

We demonstrated that E2 treatment reinstated suppressed postnatal neurogenesis in prematurely‐born rabbits. E2 supplementation led to an increase in the number of glutamatergic progenitors, Tbr2^+^ cells, at D7 and doublecortin^+^ cells at both D7 and D30. Consistent with our results, diethylstilbestrol (ER agonist) treatment in neonatal (P1) male rats results in enhanced cell survival and increased neural precursor cell (NPCs) proliferation at 14 days after the initiation of treatment.^37^ As in neonatal animals, E2 application enhances neurogenesis in the DG of adult animals in both health and disease.^38^, ^39^ Short‐term (2–4 h) exposure to E2 in adult female rats enhances neuronal proliferation in the DG, which is abolished by 48 h.^40^ In a mouse model of Alzheimer's disease, chronic administration of 17‐β estradiol enhances neurogenesis in the DJ and cognitive function.^41^ Indeed, protracted neurogenesis in the adult hippocampus preserves cognitive function in adults, whereas disrupted neurogenesis is associated with cognitive deficits.^42^ Consistent with this notion, meta‐analyses of epidemiological studies show that oestrogen replacement is considered for the treatment of Alzheimer's disease.^43^ These sets of evidence reinforce the notion that E2 replacement‐led recovery in neurogenesis would contribute to improvement in the cognitive function of premature kits.

Oestrogen plays a key role in spinogenesis and synaptogenesis. It has been suggested that E2 treatment promotes dendritic spine formation through a two‐step process.^44^ In step 1, E2 induces a transient elevation in the density of dendritic spines by increasing protein synthesis and actin polymerization. In step 2, E2 activates N‐methyl‐d‐aspartate (NMDA) receptors, resulting in the maturation and stabilization of spines.^44^ In agreement with these reports, we also found an increase in the number of glutamatergic synaptic puncta (perforant path‐granule cell synapse) in E2‐treated pups compared with vehicle controls. In addition, E2 treatment reduced the expression of PSD95, NMDA receptor‐2A (NMDAR2A) and NMDAR2B at D7, which are key regulators of dendritic spine morphology and were elevated in premature kits. As the developmental increase in PSD95 expression impedes the synaptic clustering of NR2B‐NMDARs and restricts dendritic branching,^24^ E2‐led reduction in PSD95 would contribute to spinogenesis and synaptogenesis. In the early life‐stress neonatal rat model, an elevation in NMDAR2A levels has also been reported, just as in our model of prematurity.^12^ Interestingly, the use of transactivated translational peptide (Tat) to dissociate NMDAR subunits from PSD95 rescued behavioural dysfunction.^12^ Hence, elevation in PSD95 and NMDA2A receptors obstructs synaptogenesis, and restoration in their levels would promote synaptogenesis and rescue behavioural dysfunction.

An overwhelming amount of literature suggests that E2 enhances cognitive function in both health and disease.

We demonstrated that E2 treatment improved motor as well as recognition and spatial memory of preterm kits in the present study. This enhancement in cognitive outcome can be attributed to an increase in neurogenesis and synaptogenesis. E2 protects hippocampal neurons against glutamate excitotoxicity.^45^ E2 also offers protection again oxygen‐induced apoptosis of oligodendrocytes and reverses hypoxia‐ as well as hyperoxia‐induced hypomyelination.^46^, ^47^ In addition, our previous studies have shown that oestrogen treatment restores interneuron neurogenesis in preterm newborns by cell cycle inhibition and elevation of Ascl1.^48^ Hence, E2 functions at multiple levels and in a diverse manner in brain, offering neuroprotection to both neuronal and oligodendrocytic progenitors.

**FIGURE 8:**
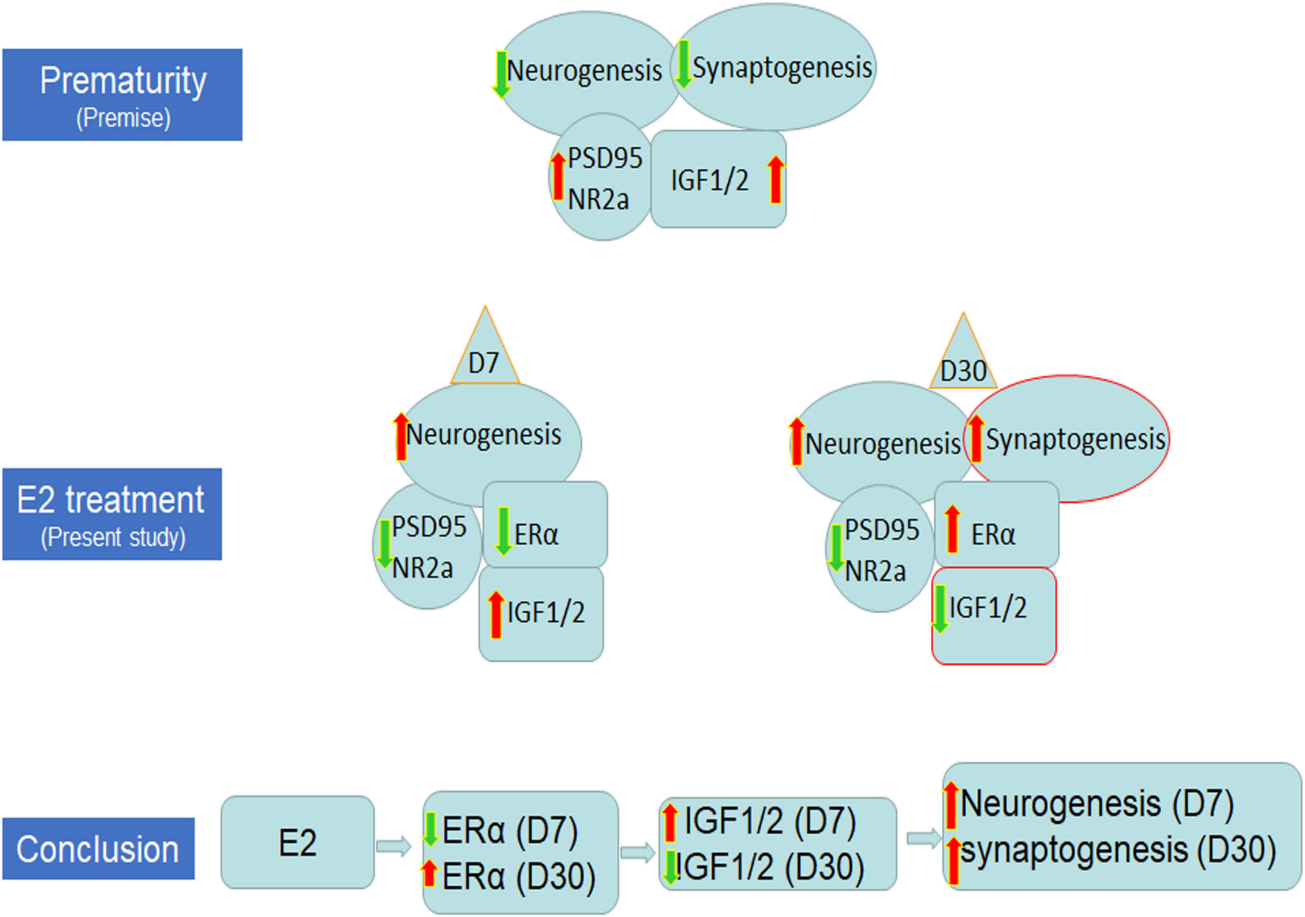
E2 regulates ERα and IGF1. (A) Our previous work shows that prematurity leads to reduced generation and accelerated maturation of neurons as well as reduced synaptogenesis, which is induced by elevated PSD95, NMDR2A and IGF1 levels. (B) In the present study, E2 treatment induced biphasic changes in oestrogen receptor α (ERα), an initial decline at postnatal day(D) 7 and subsequent elevation at D30, which was associated with reciprocal elevation (D7) and reduction (D30) in IGF1/2 expression and its downstream signalling. (C) We concluded that E2 treatment alleviates prematurity‐induced maldevelopment of DG and cognitive dysfunctions by regulating ERα and IGF1 levels.

We demonstrated that E2 treatment resulted in a biphasic response of IGF1/2 levels, an initial increase at D7 on the termination of E2 therapy, followed by decline at D30. Conversely, E2 treatment reduced ERα at D7 and elevated ERα at D30. Hence, an E2‐induced reduction in IGF1/2 was associated with an elevation in ERα. Moreover, degradation of ERα by Elacestrant treatment elevated IGF1/2 level and activated IGF signalling, suggesting IGF1 expression is negatively regulated by ERα. Implicit with our findings, previous studies have shown that ERα expression in the hippocampus varies across the estrous cycle in young adult mice, being highest when circulating oestrogen levels are lowest either after ovariectomy or during diestrus.^49^ Accordingly, chronic oestrogen treatment for 6 weeks reduced IGF1 expression in cardiac allograft transplant in male rabbits^50^ and in breast cancer disruption of oestrogen supply dramatically escalates IGF1 levels and signalling.^51^ Estradiol and IGF‐I signalling interact and exhibits harmonious action in promoting neuroprotection in models of brain injury. Estradiol and IGF synergistically activate Akt, decreasing GSK3 activity.^14^, ^15^, ^16^ However, the precise mechanistic link between IGF1 and estradiol is obscure. Our finding of estradiol downregulating ERα to activate IGF1 production at D7 with a subsequent elevation in ERα to reduce IGF1 level at D30 in the DG is novel. E2‐induced elevation in ERα at D30 might be a rebound effect of E2 withdrawal at D7 (Figure 8). We speculate that E2‐induced elevation in IGF1 promotes neurogenesis at D7 and a decline in IGF1 at D30 facilitates synaptogenesis.

The transcriptomic studies in the present study showed that genes for cell cycle, neuronal differentiation, inflammation, oxidative reaction, extracellular matrix, apoptosis, purine metabolism and HIPPO signalling pathway were significantly altered in E2‐treated kits. Consistent with our findings, hippocampal transcriptome from middle‐aged ovariectomized female rats revealed that 29 days of E2 increased the expression of genes, including IGF1, IGF2, IGFBP2, COL1A1, GPX, CLADN3 and others.^52^ In another study, E2 treatment in 10 weeks old rats during proestrus and estrous stages led to an alteration in genes regulating cell cycle, inflammation, apoptosis, extracellular matrix and neuroprotection,^53^ similar to our studies. We examined for interaction between IGF1 receptor (IGF1R) and neurodegenerative as well as glutamatergic pathway genes. Even though 19% downregulation of *IGF1R* at D30 did not pass the 49% cut‐off (fold‐change) criterion for significant downregulation, E2 treatment significantly remodelled the expression coordination of *IGF1R* with many other genes. Most notably, E2 treatment enhanced neurobehavioral function by drastically reducing (from 38.81% to 7.46%) the modulatory control of *IGF1R* on glutamatergic synaptic transmission. Moreover, E2 reduced by 25% the synergism and increased by 36% the antagonism of *IGF1R* with genes involved in amyotrophic lateral sclerosis and Alzheimer's, Huntington's, Parkinson's and prion diseases.

In this study, we employed a rabbit model reproducing the composite effect of premature birth, formula feeding and stress related to nonmaternal care to evaluate the effect of E2 on DG maldevelopment. To further understand the effect of E2 on each of the three factors, subsequent studies can be designed comparing preterm kits cared for by the foster rabbit mom vs. rabbit kits cared for by lab personnel. Importantly, preterm birth reduces plasma oestrogen concentration by 100‐fold.^54^ Accordingly, replacement oestrogen and progesterone treatment has undergone a clinical trial in extremely premature infants and has shown small benefits in improving bone mineral accretion rate and decreasing the incidence of bronchopulmonary dysplasia.^54^ These studies reinforce the value of E2 treatment in premature infants. However, E2 treatment increases the risk of thromboembolism, femininization in male children and suppression of hypothalamic–pituitary axis. E2 treatment also affects linear growth, lipid metabolism, liver function, blood pressure, neurocognition, socialization and bone and uterine health.^55^

## 5 CONCLUSION

Our study shows that 17β‐estradiol (E2) treatment reversed the prematurity‐induced reduction in neurogenesis and synaptogenesis in preterm rabbits. E2 treatment also alleviated increased PSD95 and NMDAR2A levels, thereby improving cognitive function. While E2 treatment reduced ERα at D7 and elevated ERα at D30, this led to a reciprocal elevation of IGF1 levels at D7 and reduction at D30. As degradation of ERα elevated IGF levels in the premature kits, we inferred that ERα inhibits IGF1 expression. The study identifies a novel therapeutic strategy to rescue prematurity‐related DG maldevelopment and recognizes a novel mechanism that E2 restores DG by controlling ERα and IGF1 signalling.

## AUTHOR CONTRIBUTIONS

**Deep R. Sharma:** Data curation (equal); methodology (equal). **Praveen Ballabh:** Conceptualization (equal); formal analysis (equal); funding acquisition (equal); investigation (equal); methodology (equal); project administration (equal). **Bokun Cheng:** Methodology (equal). **Rauhin Sahu:** Investigation (equal); methodology (equal). **Xusheng Zhang:** Investigation (equal); methodology (equal). **Rana Mehdizadeh:** Investigation (equal); methodology (equal). **Divya Singh:** Methodology (equal). **Dumitru Iacobas:** Data curation (equal); investigation (equal); methodology (equal).

## CONFLICT OF INTEREST STATEMENT

None.

## Supporting information


Appendix S1

## Data Availability

Data generated/analysed in the present study will be provided upon request to the corresponding author.
