# Supplementary material for: Oestrogen treatment restores dentate gyrus development in premature newborns by IGF1 regulation
Source: J Cell Mol Med. 2023 Aug 18;27(17):2467–81. doi: 10.1111/jcmm.17816 (PMC10468667; doi:10.1111/jcmm.17816)
Supplement: Supplementary file 1 — Appendix S1 [file JCMM-27-2467-s001.docx]

### SUPPLEMENTARY MATERIALS

1. **Legend to Supplemental Figures**

### Supplemental Figures 1-3

1. **Supplemental Tables 1 and 2**

### Supplementary methods

**Legend for Supplemental Figures**

**Supplemental Fig. 1: A.** *E2 treatment does not affect NeuN and GAD67 levels***.** A typical western blot analyses for GAD67 and NeuN antibodies on homogenates from E2 and vehicle treated Kits at D30. Values were normalized to ß-actin. Bar charts show means ± SEM, n = 5 each group, Student’s t-test used. Note that E2 treatment does not affect NeuN and GAD67 levels. **B.** *Estrogen treatment increases the number of calbindin interneurons and prox1 neurons in the dentate gyrus***:** Representative immunofluorescence from the DG of E2 treated kits compared to control kits at D30 labeled with Prox1 (green) and Calbindin (red) specific antibodies. Lower panel is high magnification image of the boxed area in the upper panel. Note Prox1+ and Calbindin+ cells are more abundant in E2 treated kits compared to controls. Scale bar, as indicated. Bar graphs are mean ± SEM, n=5 per group, Student’s t-test used. Inset shows low power view of the infrapyramidal blade of the DG. Stereological quantitation also shows that PV+, SST+ cells were less in E2 treated kits relative to vehicle treated kits at D30. Bar graphs are mean

± SEM, n=5 per group, Student’s t-test used.

**Supplemental Fig. 2: ERα sequences that appear to regulate IGF1: A.** Analyses of ChIP-seq datasets from human MCF7 cell line (Schmidt et al. 2010) show two ERα receptor ChIP-seq peaks--one at 2kb upstream of transcription start site for IGF1 and the other in the intron region of IGF1. **B.** Analyses of ChIP-seq datasets from mouse mammary gland (Palaniappan et al. 2019) reveal three ERα receptor Chip-seq peaks at about 40-60kb upstream of the transcription start site for IGF1. C. Rabbit genome wide motif scan for ERE against rabbit genome depict a ERE binding site at 20kb upstream of the transcriptional starting site of IGF1.

**Supplemental Fig. 3: A) E2 treatment increases the antagonism and decreases the synergism of *IGF1R* with genes involved in neurodegenerative diseases.** Statistically (p < 0.05) significant synergistically, antagonistically and independently expressed neurodegenerative diseases genes with *IGF1R* in animals treated with E2 or only with the vehicle (DMSO). B) **E2 treatment decouples *IGF1R* from the glutamatergic synapse genes.** Statistically (p < 0.05) significant synergistically, antagonistically and independently expressed glutamatergic synapse genes with *IGF1R* in animals treated with E2 or only with the vehicle (DMSO)

# A


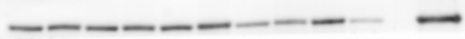
**GAD67**


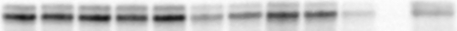
**NeuN**

**Preterm, vehicle**

**Preterm, E2**

1.5

1.0

GAD67 Adjusted OD

0.5

2.5

2.0

NeuN Adjusted OD

1.5

1.0

0.5

P=0.15

## Suppl. Fig. 1


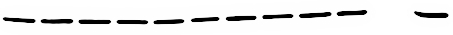
**Actin**

0.0

## Vehicle E2

0.0

## Vehicle E2

**D30**


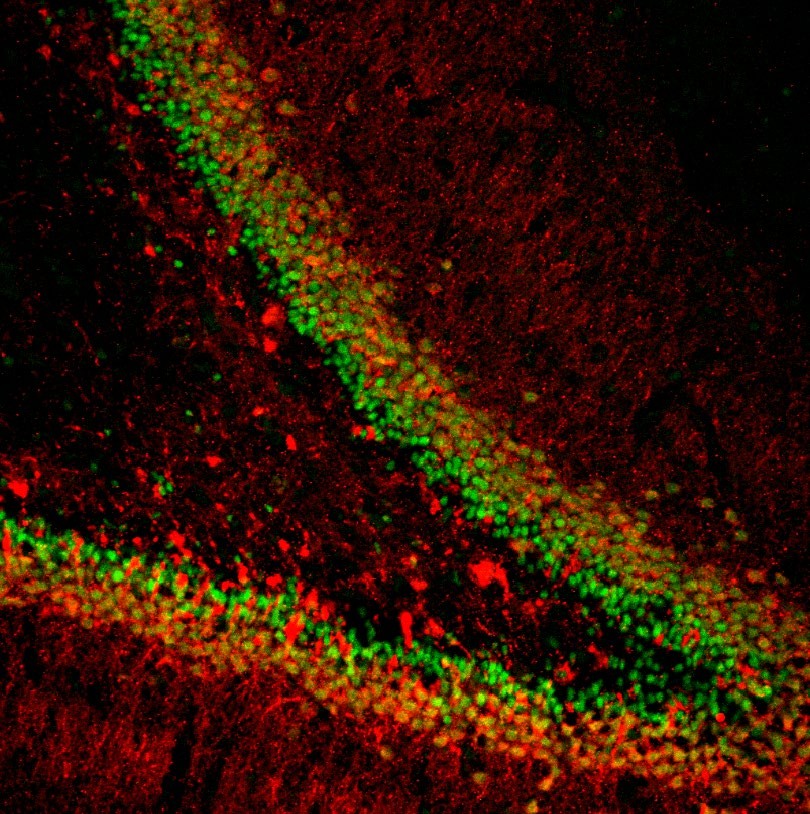

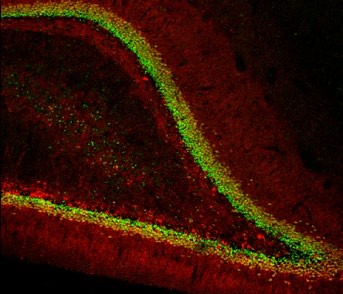


Vehicle

50µ


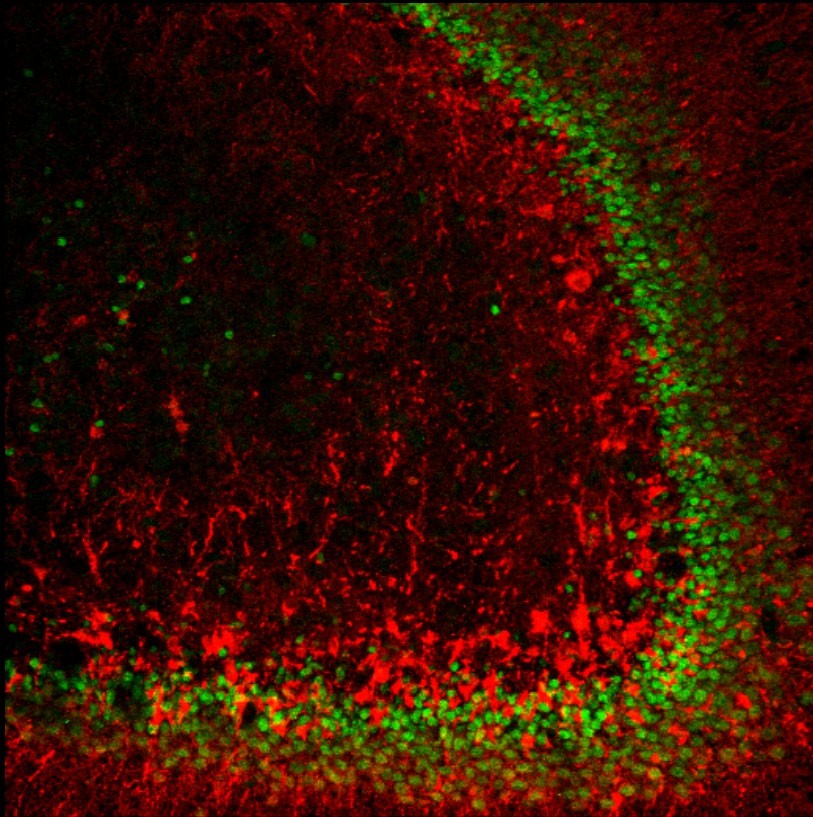

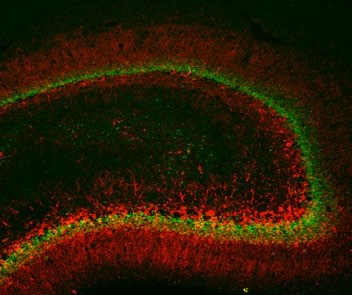


E2

50µ

B 200000

Prox1+ neurons

P=0.0206

150000

100000

Prox1 Calbindin

50000

0


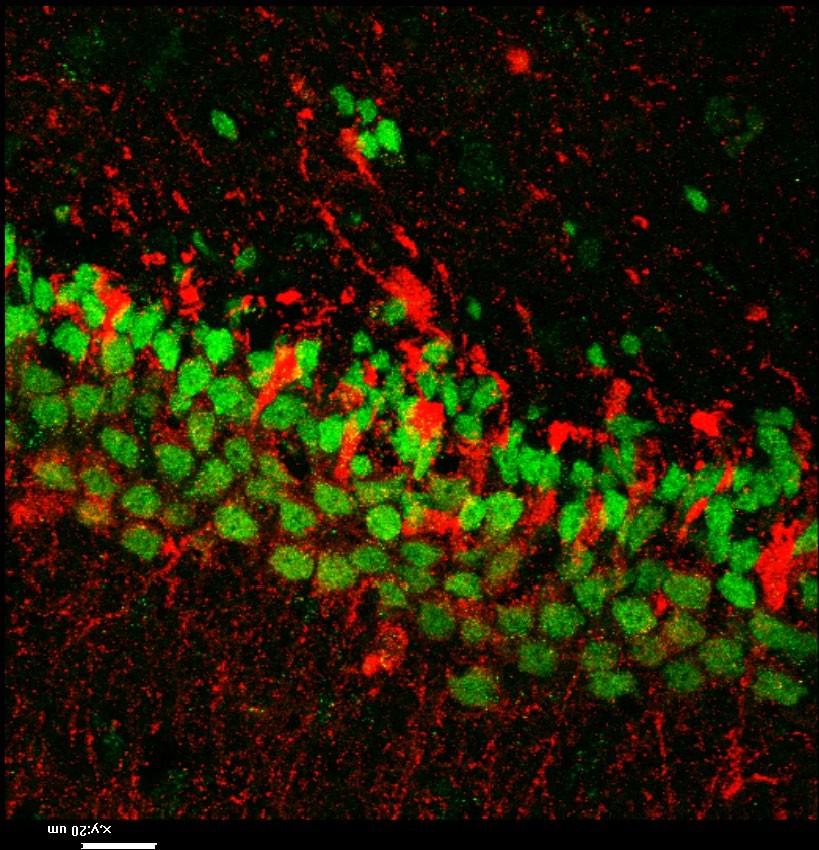


Vehicle

20µ


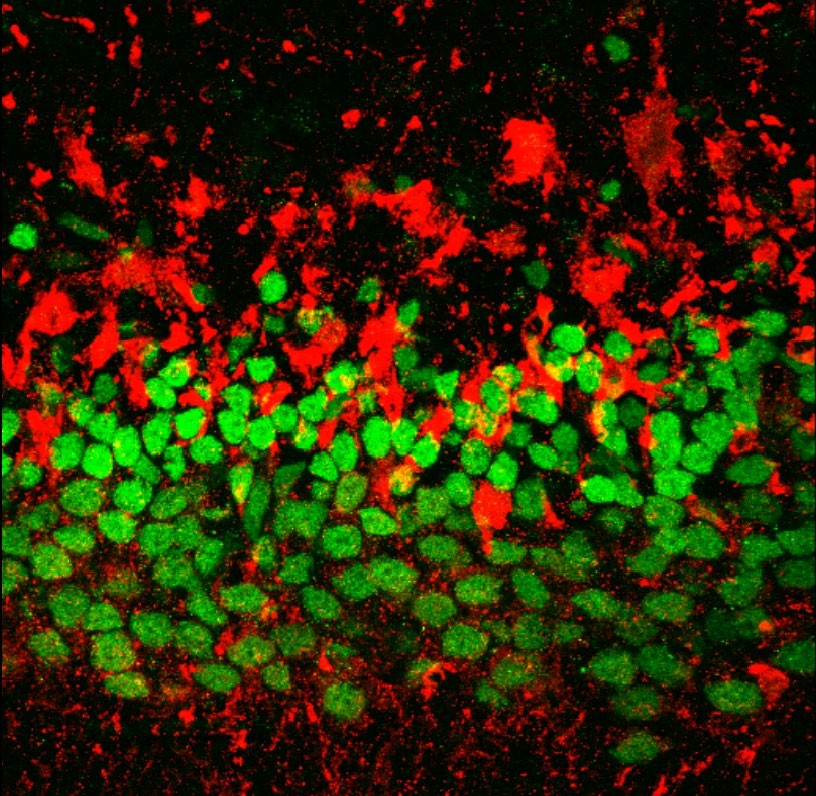


E2

20µ

20000

Calbindin+ neurons

## Vehicle E2

P=0.016

15000

10000

5000

0

## Vehicle E2


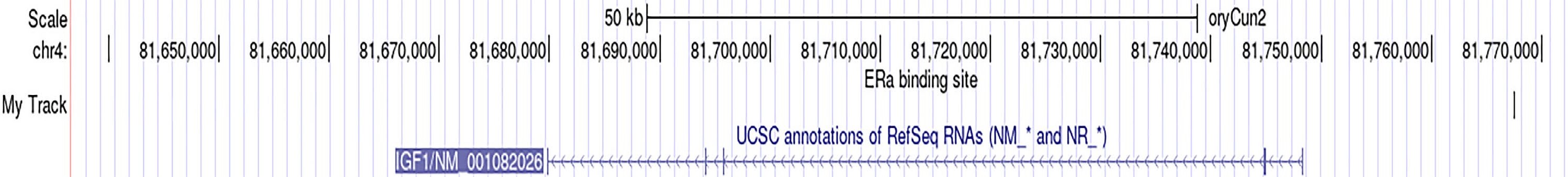
Suppl. Fig. 2


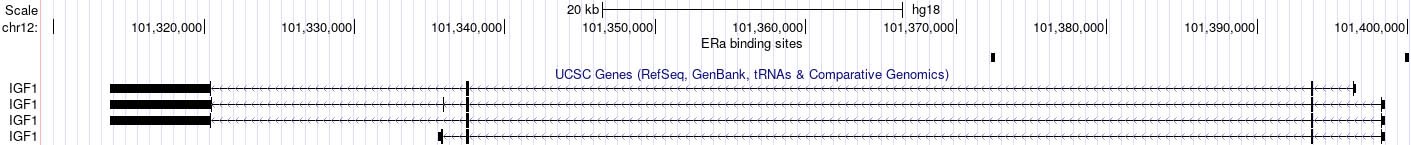
A Human


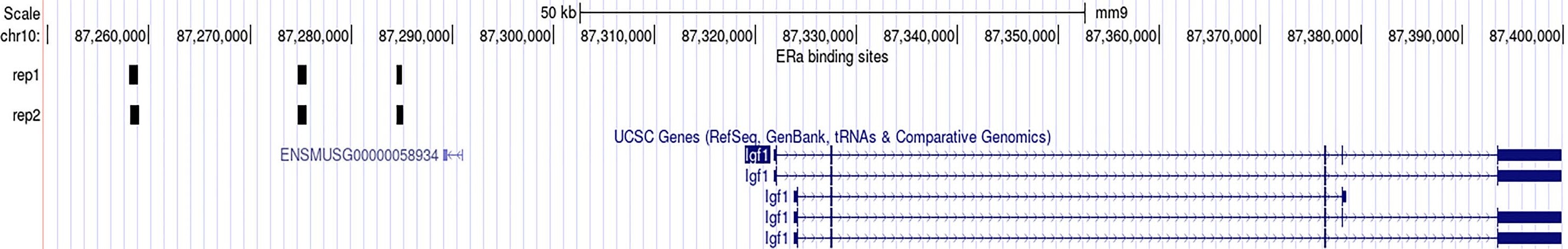
B Mouse

C Rabbit

**Suppl. Fig.3**


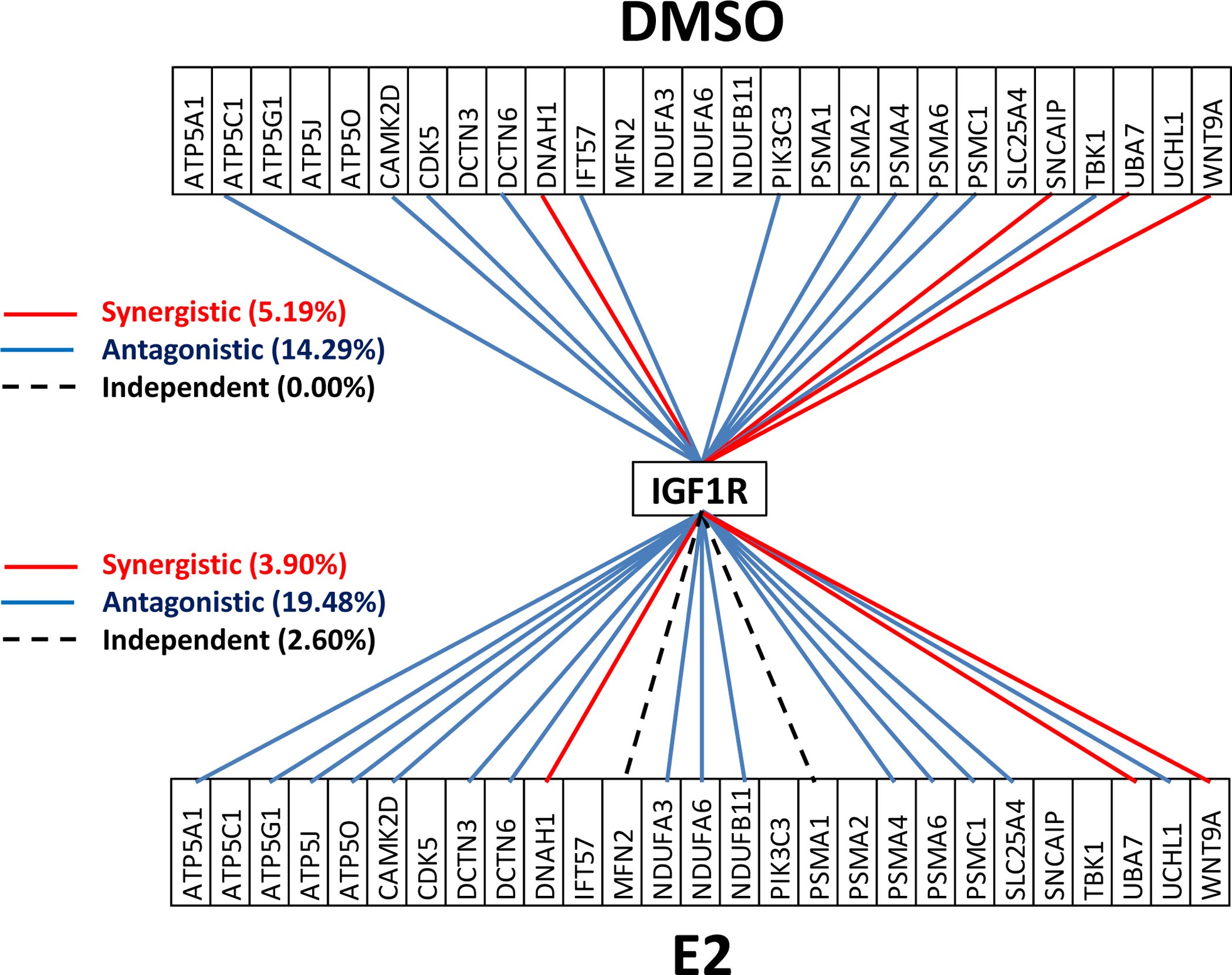

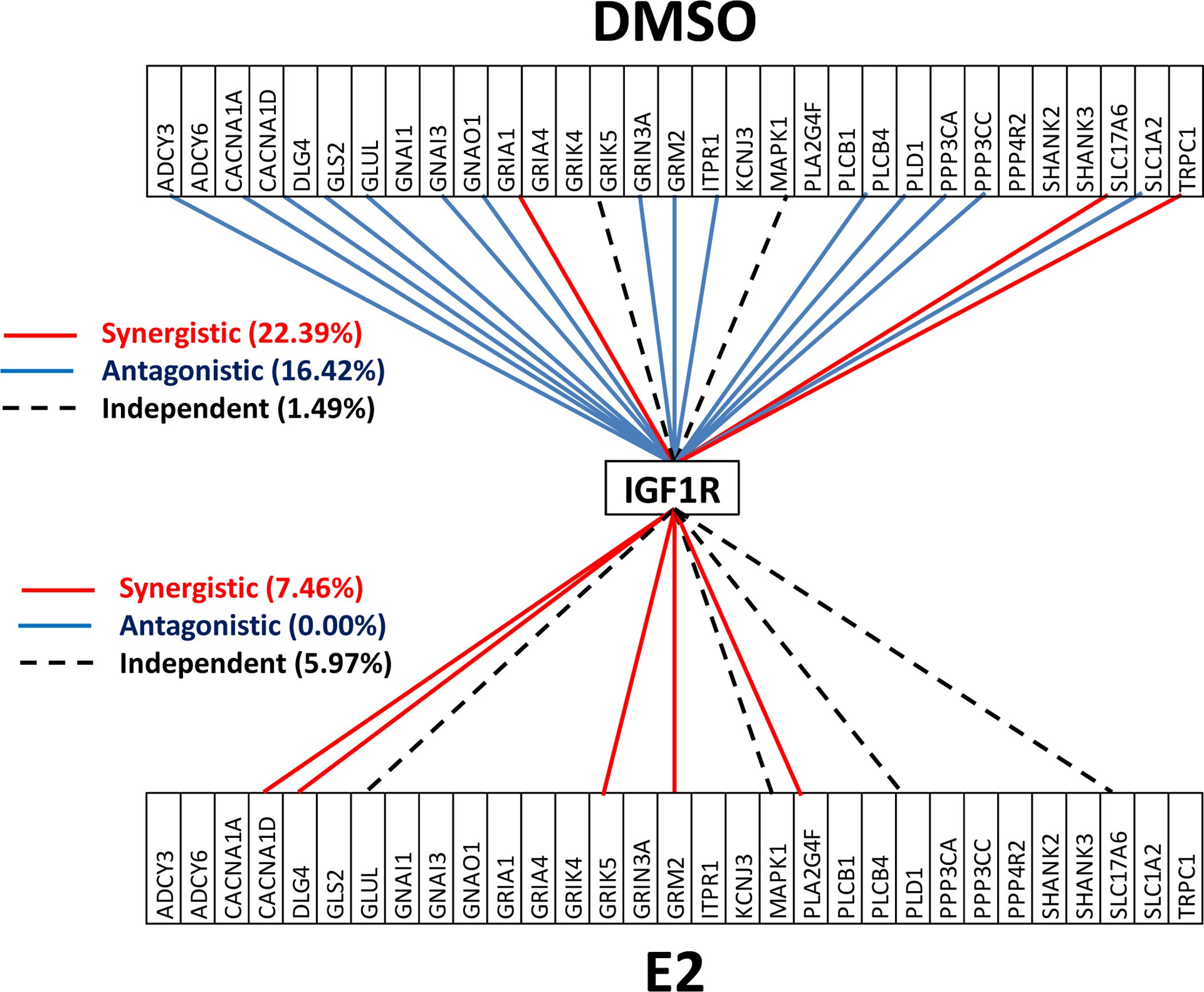


# A

B

**Supplemental Table 1: Significantly up-regulated genes.** x = expression ratio (negative for down-regulation), CUT

= absolute fold-change cut-off, WIR = Weighted Individual (gene) Regulation (negative for down-regulation).

| **Name** | **Description** | **x** | **CUT** | **WIR** |
| --- | --- | --- | --- | --- |
| AASS | aminoadipate-semialdehyde synthase | 1.83 | 1.68 | 0.08 |
| ADAMTSL1 | ADAMTS like 1 | 2.01 | 1.80 | 0.16 |
| ADCY8 | adenylate cyclase 8 | 1.84 | 1.60 | 0.20 |
| ADGB | Androglobin | 1.40 | 1.23 | 0.06 |
| ADPRH | ADP-ribosylarginine hydrolase | 2.64 | 2.62 | 0.12 |
| ALK | anaplastic lymphoma receptor tyrosine kinase | 1.72 | 1.35 | 0.16 |
| ANGPTL3 | angiopoietin like 3 | 1.78 | 1.53 | 0.17 |
| ANO4 | anoctamin 4 | 1.78 | 1.66 | 0.24 |
| ASTN2 | astrotactin 2 | 1.70 | 1.35 | 0.30 |
| AXL | AXL receptor tyrosine kinase | 2.26 | 2.08 | 0.13 |
| BYSL | bystin like | 1.38 | 1.31 | 0.22 |
| CAPN5 | calpain 5 | 1.59 | 1.35 | 0.14 |
| CAPNS1 | calpain small subunit 1 | 1.19 | 1.18 | 0.18 |
| CCDC136 | coiled-coil domain containing 136 | 1.51 | 1.50 | 0.27 |
| CCDC158 | coiled-coil domain containing 158 | 2.37 | 1.98 | 0.13 |
| CHCHD6 | coiled-coil-helix-coiled-coil-helix domain containing 6 | 1.57 | 1.34 | 0.09 |
| CLCN5 | chloride voltage-gated channel 5 | 1.60 | 1.41 | 0.09 |
| COL12A1 | collagen type XII alpha 1 chain | 1.83 | 1.65 | 0.47 |
| COL27A1 | collagen type XXVII alpha 1 chain | 2.18 | 2.18 | 0.40 |
| COL5A3 | collagen type V alpha 3 chain | 2.23 | 1.77 | 0.31 |
| CRHBP | corticotropin releasing hormone binding protein | 1.64 | 1.44 | 0.25 |
| DLX1 | distal-less homeobox 1 | 2.09 | 2.06 | 0.29 |
| DNAJC27 | DnaJ heat shock protein family | 1.24 | 1.21 | 0.13 |
| DPY30 | dpy-30, histone methyltransferase complex regulatory subunit | 1.33 | 1.19 | 0.10 |
| DPYSL5 | dihydropyrimidinase like 5 | 1.74 | 1.49 | 0.36 |
| DUSP14 | dual specificity phosphatase 14 | 1.39 | 1.36 | 0.08 |
| EEF1AKMT1 | eukaryotic translation elongation factor 1 alpha lysine methyltransferase 1 | 1.77 | 1.75 | 0.15 |
| EEFSEC | eukaryotic elongation factor, selenocysteine-tRNA specific | 1.45 | 1.41 | 0.19 |
| EFCAB11 | EF-hand calcium binding domain 11 | 1.43 | 1.31 | 0.08 |
| EPAS1 | endothelial PAS domain protein 1 | 2.79 | 2.55 | 0.24 |
| FHOD3 | formin homology 2 domain containing 3 | 1.61 | 1.54 | 0.89 |
| FIBCD1 | fibrinogen C domain containing 1 | 1.59 | 1.41 | 0.15 |
| FRAS1 | Fraser extracellular matrix complex subunit 1 | 1.70 | 1.69 | 0.64 |
| GARNL3 | GTPase activating Rap/RanGAP domain like 3 | 2.15 | 1.83 | 0.39 |
| GLB1 | galactosidase beta 1 | 1.55 | 1.43 | 0.09 |
| GSN | gelsolin | 1.82 | 1.71 | 0.26 |
| HSPG2 | heparan sulfate proteoglycan 2 | 2.33 | 1.92 | 0.18 |
| HTR2C | 5-hydroxytryptamine receptor 2C | 1.77 | 1.72 | 0.17 |
| HTRA1 | HtrA serine peptidase 1 | 1.70 | 1.56 | 0.15 |
| IFIT5 | interferon induced protein with tetratricopeptide repeats 5 | 1.79 | 1.77 | 0.08 |
| INPPL1 | inositol polyphosphate phosphatase like 1 | 1.53 | 1.48 | 0.07 |

| IQCB1 | IQ motif containing B1 | 1.36 | 1.21 | 0.13 |
| --- | --- | --- | --- | --- |
| KDSR | 3-ketodihydrosphingosine reductase | 1.25 | 1.19 | 0.19 |
| KIAA1456 | KIAA1456 ortholog | 1.35 | 1.29 | 0.09 |
| KIAA1462 | KIAA1462 ortholog | 2.08 | 1.88 | 0.38 |
| LATS2 | large tumor suppressor kinase 2 | 1.52 | 1.43 | 0.10 |
| LOC100351746 | histone H4 | 1.97 | 1.65 | 0.18 |
| LOC100355380 | HLA class I histocompatibility antigen, A-36 alpha chain | 2.38 | 1.74 | 0.18 |
| LOC100357801 | interferon-induced very large GTPase 1-like | 2.18 | 1.82 | 0.30 |
| LOC100358060 | glutamate carboxypeptidase 2 | 1.55 | 1.51 | 0.15 |
| LOC100358347 | TRPM8 channel-associated factor 2 | 1.56 | 1.51 | 0.10 |
| LOC103350157 | TATA box-binding protein-associated factor RNA polymerase I subunit D-like | 1.98 | 1.72 | 0.09 |
| MTIF3 | mitochondrial translational initiation factor 3 | 1.42 | 1.39 | 0.14 |
| NABP1 | nucleic acid binding protein 1 | 1.33 | 1.33 | 0.12 |
| NAMPT | nicotinamide phosphoribosyltransferase | 1.39 | 1.38 | 0.14 |
| NT5DC2 | 5'-nucleotidase domain containing 2 | 3.48 | 2.59 | 0.18 |
| OGDHL | oxoglutarate dehydrogenase-like | 1.82 | 1.67 | 0.25 |
| PDE5A | phosphodiesterase 5A | 2.52 | 1.96 | 0.34 |
| PDE8A | phosphodiesterase 8A | 1.49 | 1.46 | 0.09 |
| PLA2R1 | phospholipase A2 receptor 1 | 1.81 | 1.48 | 0.30 |
| PLXNB3 | plexin B3 | 1.60 | 1.35 | 0.19 |
| PPFIBP1 | PPFIA binding protein 1 | 1.56 | 1.20 | 0.23 |
| PPP1R14A | protein phosphatase 1 regulatory inhibitor subunit 14A | 1.83 | 1.69 | 0.18 |
| RAPGEF4 | Rap guanine nucleotide exchange factor 4 | 1.50 | 1.45 | 2.58 |
| RASEF | RAS and EF-hand domain containing | 1.50 | 1.41 | 0.09 |
| RHPN2 | rhophilin Rho GTPase binding protein 2 | 1.84 | 1.72 | 0.12 |
| RORA | RAR related orphan receptor A | 1.89 | 1.77 | 0.11 |
| SOX6 | SRY-box 6 | 2.76 | 2.22 | 0.18 |
| SPATA13 | spermatogenesis associated 13 | 2.18 | 1.74 | 0.19 |
| STARD13 | StAR related lipid transfer domain containing 13 | 1.55 | 1.52 | 0.20 |
| SYNE2 | spectrin repeat containing nuclear envelope protein 2 | 1.57 | 1.43 | 0.36 |
| SYT17 | synaptotagmin 17 | 1.54 | 1.35 | 0.23 |
| TEAD1 | TEA domain transcription factor 1 | 1.22 | 1.21 | 0.26 |
| TMEM243 | transmembrane protein 243 | 1.94 | 1.69 | 0.14 |
| TP53BP2 | tumor protein p53 binding protein 2 | 1.42 | 1.31 | 0.69 |
| TRUB2 | TruB pseudouridine synthase family member 2 | 1.47 | 1.47 | 0.09 |
| TTN | titin | 2.07 | 1.69 | 4.85 |
| TYRO3 | TYRO3 protein tyrosine kinase | 1.54 | 1.53 | 0.80 |
| UBASH3B | ubiquitin associated and SH3 domain containing B | 1.49 | 1.26 | 0.53 |
| WBP4 | WW domain binding protein 4 | 1.26 | 1.23 | 0.29 |
| WDR11 | WD repeat domain 11 | 1.14 | 1.13 | 0.66 |
| WNT2B | Wnt family member 2B | 1.45 | 1.42 | 0.05 |
| ZFHX4 | zinc finger homeobox 4 | 1.76 | 1.75 | 0.12 |

**Supplemental Table 2: Significantly down-regulated genes.** x = expression ratio (negative for down-regulation), CUT = absolute fold-change cut-off, WIR = Weighted Individual (gene) Regulation (negative for down-regulation).

| **Name** | **Description** | **x** | **CUT** | **WIR** |
| --- | --- | --- | --- | --- |
| ABI2 | abl interactor 2 | -1.30 | 1.27 | -2.16 |
| ALDH1L2 | aldehyde dehydrogenase 1 family member L2 | -1.16 | 1.13 | -0.30 |
| C1QTNF2 | C1q and tumor necrosis factor related protein 2 | -1.30 | 1.25 | -0.06 |
| CARD10 | caspase recruitment domain family member 10 | -1.95 | 1.88 | -0.35 |
| CCNB1 | cyclin B1 | -1.34 | 1.31 | -0.08 |
| CLN6 | ceroid-lipofuscinosis, neuronal 6, late infantile, variant | -1.45 | 1.39 | -0.20 |
| CMTM8 | CKLF like MARVEL transmembrane domain containing 8 | -1.62 | 1.46 | -0.21 |
| CNNM2 | cyclin and CBS domain divalent metal cation transport mediator 2 | -1.14 | 1.12 | -0.79 |
| COL21A1 | collagen type XXI alpha 1 chain | -1.76 | 1.54 | -1.44 |
| DBR1 | debranching RNA lariats 1 | -1.34 | 1.33 | -0.10 |
| ENTPD3 | ectonucleoside triphosphate diphosphohydrolase 3 | -1.25 | 1.22 | -0.14 |
| GALNT2 | polypeptide N-acetylgalactosaminyltransferase 2 | -1.18 | 1.17 | -0.68 |
| GPAM | glycerol-3-phosphate acyltransferase, mitochondrial | -1.23 | 1.19 | -1.37 |
| HAS3 | hyaluronan synthase 3 | -1.39 | 1.38 | -0.10 |
| IL17RB | interleukin 17 receptor B | -1.65 | 1.59 | -0.13 |
| ING4 | inhibitor of growth family member 4 | -1.36 | 1.17 | -0.20 |
| KIF9 | kinesin family member 9 | -2.07 | 1.61 | -0.33 |
| LOC100343510 | isopentenyl-diphosphate Delta-isomerase 1 | -1.83 | 1.74 | -0.30 |
| LOC100353286 | ras GTPase-activating protein-binding protein 1 | -1.86 | 1.54 | -0.28 |
| LYRM4 | LYR motif containing 4 | -1.46 | 1.45 | -0.19 |
| NARF | nuclear prelamin A recognition factor | -1.27 | 1.17 | -0.66 |
| PAQR3 | progestin and adipoQ receptor family member 3 | -1.52 | 1.37 | -0.31 |
| PDIA5 | protein disulfide isomerase family A member 5 | -1.51 | 1.49 | -0.19 |
| PHF19 | PHD finger protein 19 | -1.48 | 1.21 | -0.16 |
| PIGM | phosphatidylinositol glycan anchor biosynthesis class M | -1.46 | 1.40 | -0.06 |
| PRRT2 | proline rich transmembrane protein 2 | -1.88 | 1.83 | -1.67 |
| RBBP6 | RB binding protein 6, ubiquitin ligase | -1.18 | 1.15 | -1.06 |
| SEC22C | SEC22 homolog C, vesicle trafficking protein | -1.34 | 1.32 | -0.28 |
| SEC61A2 | Sec61 translocon alpha 2 subunit | -1.20 | 1.19 | -0.22 |
| SLC17A6 | solute carrier family 17 member 6 | -1.95 | 1.70 | -1.07 |
| SLC31A1 | solute carrier family 31 member 1 | -1.30 | 1.28 | -0.19 |
| SMTNL2 | smoothelin like 2 | -2.08 | 1.83 | -1.07 |
| SPAG5 | sperm associated antigen 5 | -2.23 | 2.03 | -0.61 |
| TMEM242 | transmembrane protein 242 | -1.46 | 1.42 | -0.49 |
| TP53INP1 | tumor protein p53 inducible nuclear protein 1 | -1.41 | 1.39 | -0.47 |
| USP4 | ubiquitin specific peptidase 4 | -1.10 | 1.07 | -0.25 |
| ZBTB2 | zinc finger and BTB domain containing 2 | -1.33 | 1.32 | -0.08 |

### Supplemental Methods

**Immunohistochemistry (IHC):** Immunohistochemical staining was performed as described in prior publication.(Panda et al., 2018) The primary antibodies used in experiments included: Mouse monoclonal Calbindin (catalog #C9848; sigma-Aldrich), guinea pig polyclonal DCX (catalog #AB2253; Millipore), rabbit Insulin growth factor -1 polyclonal (catalog #NBP2-16929; Novus Biologicals), goat polyclonal Prox1 (catalog #AF2727; Novus Biologicals), mouse monoclonal NeuN (catalog #MAB377; Millipore), rabbit monoclonal NeuN (catalog #24307S; Cell signaling), rabbit polyclonal PV (catalog #ab11427; Abcam), rat monoclonal SST (catalog #MAB354; Millipore), rabbit polyclonal PSD-95 (catalog #51-6900; Thermo Fisher Scientific), mouse monoclonal Tbr2 (catalog #14- 4877-82; eBioscience), mouse monoclonal vGlut2 (catalog #ab79157; Abcam), rabbit polyclonal GABA (catalog #A2052; Sigma-Aldrich), estrogen receptor α (CF807239), estrogen receptor β (), and mouse monoclonal Ki67 (catalog #M7240, DAKO), Secondary antibodies used were as follows: Alexa-594 conjugate donkey anti-mouse, Alexa 594- conjugate donkey anti-goat, and Alexa-488 conjugate donkey anti-rat (Jackson Immunoresearch, West Grove, PA). Briefly, we hydrated the fixed sections by immersing into 0.1M PBS, blocked the sections with normal donkey serum in PBS with 0.01% Triton-X (PBST), and incubated them with the primary antibodies diluted in PBS at 4°C overnight. After multiple washes in PBS, the sections were incubated with secondary antibody diluted in 2% normal donkey serum in PBS at room temperature for 60 minutes. Finally, after wash in PBS, sections were mounted with SlowFade Light Antifade reagent (Molecular Probes, Invitrogen, CA) and were visualized under a Confocal microscope (Nikon Instruments, Japan). Stereology was carried out using a fluorescent microscope (Axioskop 2 plus, Carl Zeiss Inc) with motorized specimen stage for automated sampling (ASI, Eugene, OR), CCD color video camera (Microfire; Optronics, Goleta, CA) and stereology software (Stereologer, SRC, Baltimore, MD). **Stereological quantification of immunolabeled images:** We counted NeuN+, Prox1+, calbindin+, GABA+, Tbr2, and Ki67+ neural cells in the immunostained sections using a Zeiss Axioskop 2 plus microscope loaded with *Stereologer hardware and software (*SRC Biosciences, Tampa, FL). Five coronal cryosections of 20 µm thickness at 100 µm intersection interval were cut from a block made of tissues fixed in 4%PFA, which were evaluated. For the quantitation NeuN+ and Prox1+ neurons, we incorporated the suprapyramidal and infrapyramidal blades of the DG within the reference space based on DAPI+ labeling, which was marked on the section under a 5x objective. The volume of the outlined area (reference space) was quantified by employing a point-counting probe (area per point 2). The number of NeuN+ and Prox1+ cells was estimated by a disector probe (frame 25 × 25μm, guard zone 2 μm, inter-frame interval 250μm) using a 60x oil lens, in which a blinded investigator clicked on cells that came into focus within the disector. For the remaining cells, the reference space included suprapyramidal blade, Infrapyramidal blade, as well as their hilus using a 5x objective. The volume of

reference space was quantified using a point-counting probe (area per point 2) as above and the number of cells was quantified by a disector probe (guard zone 2 μm, inter-frame interval 250μm) using 60x oil lens by a blinded investigator. A coefficient of error (CE) < 0.10 was taken as acceptable. This stereological quantification led to absolute values.


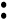

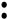
**Golgi Staining and Stereological Quantifications:** We completed Golgi staining on brain slices from preterm kits (E29) at D30 and term kits (E32 at D27 (n=5/group). The FD Rapid GolgiStain Kit (FD NeuroTechnologies, Columbia, MD) was used. Brains were immersed in a 1 1 mixture of FD Solution A B and incubated for a total of 2 weeks in the dark at room temperature. The brains were next transferred to FD Solution C and kept in the dark at 4°C for 48 hours. We next froze the brain tissues into optimum cutting temperature (O.C.T.) compound. Cryosectioning was performed on a Leica CM 3050 S cryostat at −22°C. Coronal sections were cut at 200 µm thickness and transferred to gelatin coated slides onto small drops of FD Solution C. After the sections were dry in the dark at room temperature for overnight, slides were stained, as described in the FD Rapid Golgi Stain instructions. We quantified granule cell number and dendritic branching using a Zeiss Axioskop 2 plus microscope loaded with Stereologer hardware and software *(*SRC Biosciences, Tampa, FL). The reference space, suprapyramidal and infrapyramidal blade of DG, was marked on the section under a 5x objective. The volume of the outlined area (reference space) was estimated using a point-counting probe (area per point 2). Dendrite branching was evalauted by an object area fraction probe (area per point 4, guard zone 2 μm, inter-frame interval 400μm) using 20x oil lens, in which a blinded investigator clicked on grid that overlapped the dendritic branching. The number of granule cells was counted d by a disector probe (frame 50 × 50 μm, guard zone 2 μm, inter-frame interval 250μm), in which a blinded investigator clicked on granule that came into focus within the disector area 51. A coefficient of error (CE) < 0.10 was considered acceptable. This stereological quantification of granule cell and branching led to absolute values.

**Western blot analyses:** We performed western blot analyses as previously described by us (n=5/group).(Ballabh et al., 2007) Briefly, homogenates made from hippocampus were quantified for protein by employing BCA protein assay kit (ThermoFisher Scientific, Waltham, MA). Equal amounts of protein (about 20μg) were loaded onto 4– 15% or 4–20% gradient precast gels (Bio-Rad, Hercules, CA), depending on the molecular weight of the target protein. Separated proteins were transferred onto polyvinylidene difluoride membranes using electrotransfer. Membrane was incubated overnight with primary antibodies including Mouse monoclonal Calbindin (catalog #C9848; sigma-Aldrich), rabbit monoclonal DCX (catalog #4604S; Cell signaling), rabbit polyclonal GFAP (catalog #AB5804; Millipore), rabbit monoclonal NeuN (catalog #24307P; Cell signaling), mouse monoclonal β-Actin (catalog #A5316; Sigma-Aldrich), rabbit polyclonal PSD-95 (catalog #51-6900; Thermo Fisher Scientific), rabbit

IGF1Rβ, p-IGF-1Rβ, pmTOR, pAKT, pGSK3β, pFOXO1, pTuberin, Cell signaling kit (catalog #42022T; Cell signaling), rabbit polyclonal Akt (catalog #9272S; Cell signaling), rabbit monoclonal GSK-3beta (catalog #9315S; Cell signaling), mouse monoclonal NMDAR1 (catalog #32-0500; Thermo Fisher Scientific), rat polyclonal NMDAR2A (catalog #A-6473; Thermo Fisher Scientific), rabbit polyclonal NMDAR2B (catalog #71-8600; Thermo Fisher Scientific), goat polyclonal IGF-II (catalog #AF-292NA; Novus Biologicals), rabbit polyclonal IGF-1 (catalog #NBP2-16929; Novus Biologicals), rabbit polyclonal IGFBP-3 (catalog #10189-2-AP; Proteintech), goat polyclonal Human growth hormone (catalog #AF1067-SP; Novus Biologicals), rabbit monoclonal β-catenin (catalog #8480S; Cell signaling), rabbit monoclonal GSK-3β (catalog #9315S; Cell signaling), mouse monoclonal GFAP (catalog #G3893; Sigma-Aldrich), rabbit polyclonal p-NMBAR2B (catalog #4208S; Cell signaling) and rabbit monoclonal p- IGF1R beta (catalog #80732S; Cell signaling) and target proteins were detected by an ECL system by using secondary antibodies conjugated with horseradish peroxidase (1:2000 HRP antibody, Jackson Immuno-research, West Grove, PA). The blots from every experiment were run two to three times and were densitometrically analyzed using ImageJ (NIH, Bethesda, MD). Optical density values were normalized to β-actin.

### Isolation of Nuclei and FACS sorting of hippocampal dentate gyrus granule cells

Freshly dissected dentate gyrus of full term and preterm kit brain tissue was homogenized in cold lysis buffer (0.32M Sucrose, 5 mM CaCl2, 3 mM Magnesium acetate, 0.1 mM, EDTA pH 8.0, 10mM Tris-HCl, pH8, 1 mM DTT, 0.1% Triton X-100) and 0.4 U µl−1 recombinant RNase inhibitor (Clontech/Takara, Cat. # 2313A) using a Wheaton Dounce tissue grinder (40-50 strokes with the loose pestle) and filtered via a 40µm cell strainer. The flow-through was underlaid with 1.8M sucrose buffer (1.8 M Sucrose, 3 mM Magnesium acetate, 1 mM DTT, 10 mM Tris-HCl pH8.0, 0.12 U µl−1 recombinant RNase inhibitor (Clontech/Takara, Cat. # 2313A)) gradient and ultracentrifuged at 24,000 rpm for 1 hour at 4°C using an SW32 rotor in order to remove brain myelin, fat and debris. The pellets were next re-suspended in the blocking buffer (PBS incubated in 0.1% final BSA concentration) containing primary antibody and/or conjugated with secondary antibody and incubated for about 1.5 hours. For isolation of dentate gyrus granule cells, we used anti-NeuN antibody (1:1000, Alexa488 conjugated, Millipore Cat #MAB377X), anti- Prox1(1:1000, Dylight 680, Novus Cat #NBP2-77071FR) and Prior to FACS sorting, DAPI (Thermoscientific, USA) was added to a final concentration of 1µg/ml. GFP+, Prox1+ and NeuN+ granule cell nuclei were sorted into individual tubes (pre-coated with 5% BSA) containing 300 µl RNA extraction buffer per 100,000 cells using a BD FACSAria™ High Sensitivity Flow Cytometer.

### RNA seq and bioinformatics analyses:

RNA extraction, library preparations, sequencing reactions and bioinformatic analysis were conducted by GENEWIZ, LLC. (South Plainfield, NJ, USA). RNA samples were extracted with Trizol (Invitrogen, Carlsbad, CA)

as in manufactures’ instructions. Extracted samples were quantified using Qubit 2.0 Fluorometer (Life Technologies, Carlsbad, CA, USA) and RNA integrity was assessed using the RNA Screen Tape on Agilent 2200 TapeStation (Agilent Technologies, Palo Alto, CA, USA). RNA sequencing libraries were prepared with SMARTer® Stranded Total RNA-Seq Kit - Pico Input Mammalian kit as in manufacturer’s protocol (Takara, Cat. # 634411). Sequencing libraries were validated by DNA Analysis Screen Tape on the Agilent 2200 TapeStation (Agilent Technologies, Palo Alto, CA, USA), and quantified by using Qubit 2.0 Fluorometer (Invitrogen, Carlsbad, CA) as well as by quantitative PCR (KAPA Biosystems, Wilmington, MA, USA). The pooled libraries were clustered on a flowcell. After clustering, the flowcell was loaded on the Illumina HiSeq instrument (4000 or equivalent) as in manufacturer’s instructions and sequenced using a 2x150 bp Paired End (PE) configuration. Image analysis and base calling were carried out by the HiSeq Control Software (HCS). Raw sequence data produced from Illumina HiSeq was converted into Fastq files and de-multiplexed using Illumina's bcl2fastq 2.17 software. One mismatch was permitted for index sequence identification.

After investigating the quality of the raw data, sequence reads were trimmed to eliminate possible adapter sequences and nucleotides with poor quality using Trimmomatic v.0.36. The trimmed reads were plotted to the *Rabbit Oryctolagus* reference genome available on ENSEMBL using the STAR aligner v.2.5.2b. The STAR aligner is a splice aligner that detects splice junctions and includes them to help align the entire read sequences. BAM files were created as a result of this step. Unique gene hit counts were calculated by using feature Counts from the Subread package v.1.5.2. Only unique reads that dropped within exon regions were counted.

A In order to minimize the effect of the technical noise, all genes with less than counts were eliminated from the analysis and the rest were normalized to the median expression in each sample. The arbitrarily introduced absolute log2 (fold-change) >1 could be too stringent for stably expressed genes or too relaxed for highly variably expressed genes. Therefore, previously published algorithm was used to determine for each gene the absolute fold-change cut-off to consider it as significantly regulated. We determined also the Weighted Individual (gene) Regulation that considers the total change of the expression level multiplied by the statistical confidence in this change to better assess the contribution of that gene to the overall transcriptomic regulation. PCA analysis was achieved using the "plotPCA" function within the DESeq2 R package. The plot shows the samples in a 2D plane covered by their first two principal components. The top 500 genes, selected by highest row variance, were used to create the plot.
